# The Beta Subunit of Nascent Polypeptide Associated Complex Plays A Role in Flowers and Siliques Development of *Arabidopsis thaliana*

**DOI:** 10.3390/ijms21062065

**Published:** 2020-03-17

**Authors:** Jan Fíla, Božena Klodová, David Potěšil, Miloslav Juříček, Petr Šesták, Zbyněk Zdráhal, David Honys

**Affiliations:** 1 Laboratory of Pollen Biology, Institute of Experimental Botany of the Czech Academy of Sciences, 16502 Praha 6, Czech Republic; klodova.bozena@gmail.com (B.K.); david@ueb.cas.cz (D.H.); 2 Department of Experimental Plant Biology, Faculty of Science, Charles University, 12800 Praha 2, Czech Republic; 3 Mendel Centre for Plant Genomics and Proteomics, Central European Institute of Technology, Masaryk University, 62500 Brno, Czech Republic; david.potesil@ceitec.muni.cz (D.P.); zdrahal@sci.muni.cz (Z.Z.); 4 Station of Apple Breeding for Disease Resistance, Institute of Experimental Botany of the Czech Academy of Sciences, 16502 Praha 6, Czech Republic; juricek@ueb.cas.cz; 5 Laboratory of Functional Genomics and Proteomics, National Centre for Biomolecular Research, Faculty of Science, Masaryk University, 62500 Brno, Czech Republic

**Keywords:** *Arabidopsis thaliana*, chaperone, flower bud proteome, flower bud transcriptome, male gametophyte, nascent polypeptide-associated complex

## Abstract

The nascent polypeptide-associated (NAC) complex was described in yeast as a heterodimer composed of two subunits, α and β, and was shown to bind to the nascent polypeptides newly emerging from the ribosomes. NAC function was widely described in yeast and several information are also available about its role in plants. The knock down of individual NAC subunit(s) led usually to a higher sensitivity to stress. In *Arabidopsis thaliana* genome, there are five genes encoding NACα subunit, and two genes encoding NACβ. Double homozygous mutant in both genes coding for NACβ was acquired, which showed a delayed development compared to the wild type, had abnormal number of flower organs, shorter siliques and greatly reduced seed set. Both *NACβ* genes were characterized in more detail—the phenotype of the double homozygous mutant was complemented by a functional NACβ copy. Then, both *NACβ* genes were localized to nuclei and cytoplasm and their promoters were active in many organs (leaves, cauline leaves, flowers, pollen grains, and siliques together with seeds). Since flowers were the most affected organs by *nacβ* mutation, the flower buds’ transcriptome was identified by RNA sequencing, and their proteome by gel-free approach. The differential expression analyses of transcriptomic and proteomic datasets suggest the involvement of NACβ subunits in stress responses, male gametophyte development, and photosynthesis.

## 1. Introduction

The nascent polypeptide-associated complex (NAC) is present in archaea and eukaryotes, and on the contrary absent from bacteria [1]. The abbreviation NAC should not be confused with the family of transcription factors, called also NAC. Throughout this manuscript, the mentioned abbreviation NAC will be used in the meaning of nascent polypeptide-associated complex. In eukaryotes, the NAC complex was described as a heterodimer composed of an α- and a β-subunit [1] whereas archaeal NAC was represented by an α-homodimer [2]. By now, the roles of NAC complex were mainly studied in yeast and animals [3]. Yeast genome encodes for two *NACβ* homologues (named β1, and β3), and a single *NACα* gene. The yeast NACβ proteins differed in their affinity to the α-subunit since the α/β1 complex was significantly more abundant than the alternative dimer α/β3 [4]. Beside this, both isoforms of the NAC complex play likely distinct roles since the function of SSb chaperone complex was complemented by both individual isoforms of NAC complex with a different efficiency [5]. NAC complex formed by α/β heterodimer usually binds to the newly synthesized nascent polypeptides emerging from the ribosome, and thus functions as a chaperone [6,7]. It is able to bind proteins in various conformations, such as unfolded, folded, or intrinsically disordered ones [8], and likely interacts with the ribosome via the C-terminal domain of the β-subunit [9]. Moreover, NAC complex has likely more functions on the cellular level, for instance in protein sorting to endoplasmic reticulum [7,10] or to mitochondrion [11]. Nevertheless, the role of NAC complex in protein sorting to mitochondria was observed exclusively in vitro but not in vivo. Thus, the role of NAC complex in protein sorting in yeast is not yet confirmed on the molecular level. However, the role of NAC complex in protein sorting was proven in *Caenorhabditis elegans* where it blocked the ribosome binding site on the endoplasmic reticulum in case the protein should reside in the cytoplasm [12]. Besides its function as a chaperone and in protein sorting, NAC was also hypothesized to play a role in ribosome biogenesis [13]. In higher eukaryotes, the NAC complex was essential for embryo development since its knock down caused embryonic lethality in mouse, nematode and drosophila [14,15,16]. Until now, the function of NAC subunits forming the heterodimeric complex was considered but they can both play their roles also individually as single subunits. The NACβ subunit is also known as basic transcription factor 3 (BTF3) [15,16,17], whereas the sole α-subunit works as a transcriptional co-activator [18,19].

Several information about the role of the NAC complex are also known in plants. The complexity of the formed heterodimer variants could be even higher in *Arabidopsis thaliana* since its genome encodes for five NACα genes (NACα1—At3g12390, NACα2—At3g49470, NACα3—At5g13850, NACα4—At4g10480, NACα5—At1g33040) and two NACβ homologues (NACβ1—At1g73230, and NACβ2—At1g17880) according to the TAIR database [20]. Arabidopsis NACβ1, and NACβ2 (named in the study as BTF3, and BTF3L) were reported to play their roles in cold stress, during which were phosphorylated by OST1 [21]. NACα2 was reported to interact with phytochromes in the cytoplasm [22], and NACβ was shown to interact with eIF(iso)4E [23]. The other plant studies regarding NAC complex or its subunits were carried out on different species than *Arabidopsis thaliana*. Firstly, the NAC role in plant development was reported. NACβ in *Nicotiana benthamiana* was shown to play its role during leaf development since its leaves showed an abnormal morphology with reduced chloroplast size and chlorophyll content after NACβ silencing [24]. A similar NACβ function was reported in wheat *(Triticum aestivum)* [25] and a role in seed germination and seedling growth was shown in rice *(Oryza sativa)* [26]. Secondly, NAC complex plays a key role under stress conditions, particularly during drought or salt stress in barley *(Hordeum marinum)* [27] and in transgenic *A. thaliana* overexpressing *NAC* genes from *Spartina alterniflora* [28]. NACβ by itself was also shown to be responsible for tolerance to freeze and drought stress in wheat *(Triticum aestivum)* [29], and for flooding tolerance in soybean *(Glycine max)* [30]. Not only abiotic stress but also NACβ role during hypersensitive response to pathogens in hot pepper *(Capsicum annuum)* [31] was reported. Last but not least, in *Nicotiana benthamiana*, the role of NACα in cell-to-cell movement of Brome mosaic virus (BMV) was presented [32]. 

Herein, we investigated the role of NACβ subunits in the flowers and siliques development of *Arabidopsis thaliana*; we characterized both *NACβ* genes (complementation analysis, overexpression, subcellular localization, promoter activity) and identified and characterized flower bud transcriptome and proteome of double homozygous mutant plants in both *NACβ* genes, suggesting the likely role of NACβ proteins in stress responses, male gametophyte development, and photosynthesis.

## 2. Results

### 2.1. *nacβ1nacβ2* Double Homozygous Plants Have Abnormal Flowers and Reduced Seed Set

**Figure 1:**
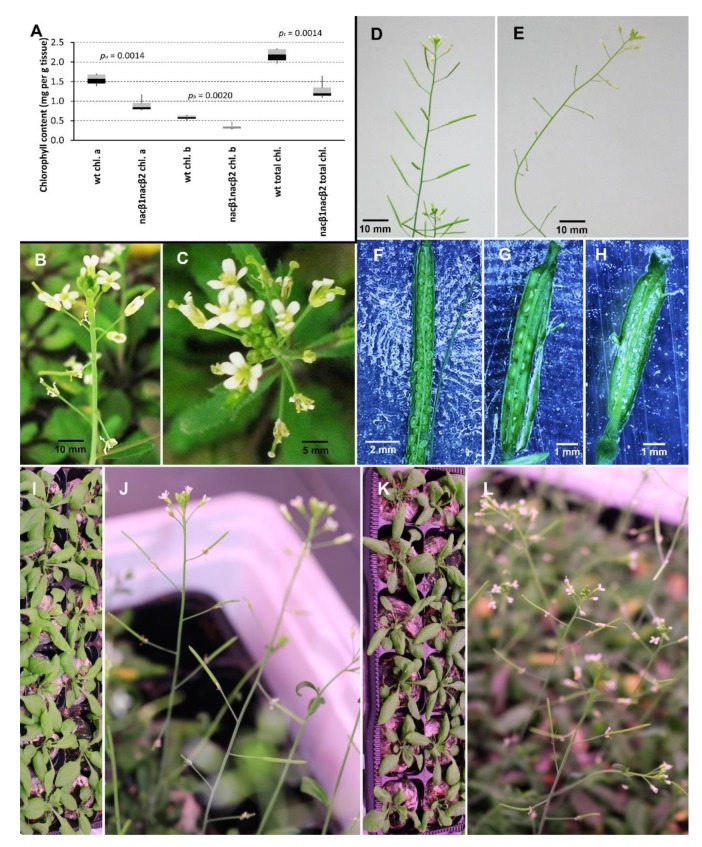
Phenotype defects of the *nacβ1nacβ2* plants and their complementation. (**A**)—Box-and-whisker plot showing the median and quartiles from four independent experiments revealing the chlorophyll a, chlorophyll b, and total chlorophyll content in the *nacβ1nacβ2*, and Col-0 wild type (wt) leaves. The obtained values of chlorophyll content in *nacβ1nacβ2*, and Col-0 were compared by Student’s t-test. The calculated *p*-values of three comparisons are given: *p_a_*—chlorophyll a; *p_b_*—chlorophyll b; *p_t_*—total chlorophyll. (**B**)—Col-0 wt inflorescence with normally looking flowers. Scale bar represents 10 mm. (**C**)—*nacβ1nacβ2* inflorescence with flowers containing different number of floral organs. Scale bar represents 5 mm. (**D**)—Col-0 wt siliques of a normal size with a normal seed set. Scale bar represents 10 mm. (**E**)—*nacβ1nacβ2* siliques, which were significantly shorter. Scale bar represents 10 mm. (**F**)—Col-0 wt seeds inside unripen silique, most of the seeds are normal-sized and green. Scale bar represents 2 mm. (**G**)—*nacβ1nacβ2* seeds inside the silique of less severe phenotype, several normal-sized and green seeds are combined with aborted seeds. Scale bar represents 1 mm. (**H**)—*nacβ1nacβ2* seeds inside the silique of more severe phenotype where most seeds are aborted. Scale bar represents 1 mm. (**I**,**J**)—*nacβ1nacβ2* plants complemented with proNACβ1::NACβ1–GFP construct. I—Plants with leaf rosettes, 27 day after seed sowing. J—Flowering plants 36 days after seed sowing. (**K**,**L**)—*nacβ1nacβ2* plants complemented with proNACβ2::NACβ2–GFP construct. K—Plants with leaf rosettes, 27 day after seed sowing. L—Flowering plants 36 days after seed sowing.

The seeds of *Arabidopsis thaliana* T-DNA insertion lines bearing the insertion in each of the two genes coding for NACβ (SALK_043673 in *NACβ1*—*nacβ1* and GK-368_H02 in *NACβ2* –*nacβ2*; the lower-case letters in italics represent a homozygous mutant) were sown, and subsequently conventionally genotyped by polymerase chain reaction (PCR) combining gene-specific and insert-specific primer pairs. None of the studied homozygous mutants showed any significant changes in their phenotypic traits (such as whole plant habitus, and various organ traits, including mature pollen) when compared to the Columbia-0 wild type (Col-0 wt) plants. The two mentioned T-DNA insertion lines were crossed with each other (Figure S1D,E). According to quantitative reverse transcriptase polymerase chain reaction (qRT-PCR) data, the SALK_043673 line represented a knockout, whereas GK-368_H02 carried knocked down target gene (Figure S1F). The first generation after the cross consisted of heterozygous plants in both studied genes (*nacβ1/+;nacβ2/+*), and by a series of self-crosses, the double homozygous mutant in both *NACβ* genes (*nacβ1nacβ2*) was acquired. The genotype of the crossed plants and their offspring was determined by a set of conventional PCR reactions combining gene-specific and insert-specific primer pairs. Exclusively, the *nacβ1nacβ2* double homozygous plants showed phenotypic defects compared to the Col-0 wt plants as follows (Figure 1; Figure S1A–C; Figure S2). (i) The *nacβ1nacβ2* plants were delayed in their development for 10–14 days compared to the control Col-0 wt plants. Although the *nacβ1nacβ2* germinated only 1–3 days later or nearly at the same time as the Col-0 wt plants did, they lagged longer (10–14 days) during stem formation, setting the first flowers, setting the first siliques, and finally starting seed maturation (Figure S1A–C). (ii) The average chlorophyll content in the *nacβ1nacβ2* leaves (1.28 mg total chlorophyll per g tissue) represented only three fifths of the Col-0 wt leaves (2.17 mg total chlorophyll per g tissue) (Figure 1A). However, the ratio of chlorophyll a to chlorophyll b remained practically the same (2.62 or 2.65, respectively). (iii) Around 90% flowers of the *nacβ1nacβ2* showed abnormal number of flower organs, mostly 5–6 sepals, 5–6 petals, and 7 anthers but there was not only one distinct phenotype category but several of them (Figure 1B,C). The most abundant category (28% total flowers) was represented by flowers with 5 sepals, 5 petals, and 6 anthers followed by 12% flowers with 5 sepals, 5 petals, and 7 anthers (Figure S2A). Furthermore, the gynoecia in notable part of flowers were not grown together. (iv) The *nacβ1nacβ2* siliques were significantly shorter than the Col-0 wt ones; they reached in average only 5 mm (Figure 1E, Figure S2B) compared to the Col-0 wt average of 14 mm (Figure 1D, Figure S2B). (v) The phenotypic characteristics were obvious also inside the siliques: the *nacβ1nacβ2* plants produced significantly less seeds (median of total seeds per silique reached 15, whereas the Col-0 wt had the median of 53 seeds per silique, Figure S2C). Furthermore, the proportion of the normal-sized and -coloured seeds in the *nacβ1nacβ2* siliques reached 14–23% (Figure 1G,H, Figure S2D) since most of the seeds were represented by the aborted embryo sacks that did not undergo fertilization. On the other hand, the Col-0 wt siliques were filled with 98% viable seeds (Figure 1F, Figure S2D).

### 2.2. *nacβ1nacβ2* Double Mutation Compromises Progamic Phase and Ovule Targeting

**Figure 2:**
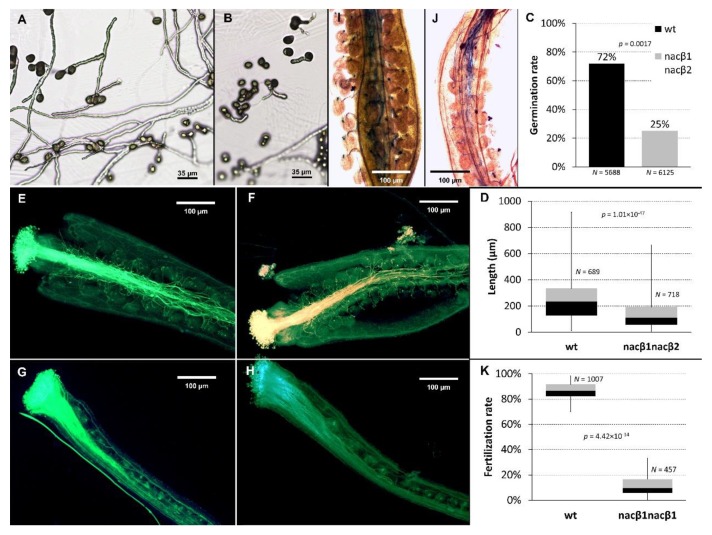
Functional tests revealing the causes of the *nacβ1nacβ2* phenotype. (**A**)—Col-0 wt pollen tubes cultivated 8 hours in vitro. Scale bar represents 35 µm. (**B**)—*nacβ1nacβ2* pollen tubes cultivated 8 hours in vitro. Scale bar represents 35 µm. (**C**)—Column chart showing average germination efficiency in three independent experiments of the Col-0 wt pollen and *nacβ1nacβ2* pollen under in vitro conditions. Both datasets were statistically compared by Student’s t-test, p-value of which is given. (**D**)—Box-and-whisker plot showing the median and quartiles of pollen tube length from three independent experiments germinating Col-0 wt pollen and *nacβ1nacβ2* pollen in vitro. Both datasets were statistically compared by Student’s t-test, p-value of which is given. (**E**)—Aniline blue staining of Col-0 wt pistils in vivo pollinated with Col-0 wt pollen. Scale bar represents 100 µm. (**F**)—Aniline blue staining of Col-0 wt pistils in vivo pollinated with *nacβ1nacβ2* pollen. Scale bar represents 100 µm. (**G**)—Aniline blue staining of *nacβ1nacβ2* pistils in vivo pollinated with Col-0 wt pollen. Scale bar represents 100 µm. (**H**)—Aniline blue staining of *nacβ1nacβ2* pistils *in vivo* pollinated with *nacβ1nacβ2* pollen. Scale bar represents 100 µm. (**I**)—The blue dot assay showing the fertilization efficiency of Col-0 wt pistil by labelling the pollen tubes with proLAT52–GUS. Every fertilization event is represented by a blue dot. Scale bar represents 100 µm. (**J**)—The blue dot assay showing the fertilization efficiency of *nacβ1nacβ2* pistil by labelling the pollen tubes with proLAT52–GUS. Scale bar represents 100 µm. (**K**)—Box-and-whisker plot showing the median and quartiles of fertilization rate of *nacβ1nacβ2* and Col-0 wt pistils in three independent experiments. The embryo sacs marked in blue were considered as fertilized whereas the unstained ones were regarded as unfertilized. Both datasets were statistically compared by Student’s t-test, p-value of which is given.

Since the *nacβ1nacβ2* mature pollen grains did not show any developmental defects compared to the Col-0 wt as described in Reňák et al. (2011) [33], the processes in male gametophyte development and in pollen tube growth and targeting that underly the formation of *nacβ1nacβ2* phenotype were tested. (i*) In vitro pollen tube cultivation.* Under the in vitro conditions [34], *nacβ1nacβ2* pollen grains germinated less efficiently compared to Col-0 wt since only, on average, 25% *nacβ1nacβ2* pollen grains germinated (Figure 2B,C). On the other hand, the Col-0 wt pollen tubes showed average 72% germination (Figure 2A,C). Moreover, *nacβ1nacβ2* pollen grains showed not only a reduced germination rate but also pollen tubes after 8 h cultivation were shorter than the Col-0 wt ones. The *nacβ1nacβ2* pollen tubes reached average 142µm, whereas the control Col-0 wt pollen tubes reached almost double average length of 252µm (Figure 2D). These data suggested that pollen germination together with pollen tube growth were affected in the *nacβ1nacβ2* plants. However, the pollen germination in vitro can be different from in vivo since the cultivation in vitro lacks female gametophyte cues, which strongly influence the behavior of male gametophyte [35]. (ii) *Aniline blue staining of the in vivo-grown pollen tubes.* To address this limitation of the in vitro pollen tube cultivation, the pollen grains were germinated again, this time in vivo. The flower buds to be pollinated were emasculated the day before, and the pollen grains were let to germinate on their pistils. After the overnight growth, the callose in the cell wall of the grown pollen tubes was stained by aniline blue [36]. The *nacβ1nacβ2* embryo sacs were less efficient in attracting the Col-0 wt pollen tubes (Figure 2F) compared to the Col-0 wt embryo sacs (Figure 2E). Similarly, the *nacβ1nacβ2* pollen tubes grew more slowly and were less efficiently attracted by the Col-0 wt embryo sacs (Figure 2G). In case of *nacβ1nacβ2* pistils pollinated with *nacβ1nacβ2* pollen grains, the targeting efficiency of the pollen tubes was dramatically reduced (Figure 2H). The in vivo pollen tube growth verified that *nacβ1nacβ2* pollen tube growth was negatively influenced and suggested that *nacβ1nacβ2* showed also defects in female gametophyte. (iii*) Blue dot assay.* Aniline blue staining of the *in vivo* grown pollen tubes provides with information about ovule targeting but does not enable quantification of the targeted versus untargeted ovules. To score the fertilization events, blue dot assay was performed [37]. By calculating the proportion of targeted embryo sacs by wt pollen tubes (carrying the proLAT52–GUS construct), it was possible to decipher the targeting efficiency, which reached in case of *nacβ1nacβ2* pistils 11 % (Figure 2J,K) whilst in case of Col-0 wt pistils was much higher, 82% (Figure 2I,K).

### 2.3. *nacβ1 and nacβ2* Alleles Show Partly Reduced Transmission Efficiency

So far, the phenotype of the *nacβ1nacβ2* plants was described together with the nature of phenotypic traits in male gametophyte development, pollen tube growth and targeting efficiency. However, it was also necessary to test the transmission efficiency of the *nacβ* mutant alleles to the next generation and compare it with the wild type *NACβ* alleles. To achieve this, a set of two self-crosses, and eight out-crosses was performed. Since there are two *NACβ* genes in *Arabidopsis thaliana* genome, the genotypic background of the second gene was considered. At first, plants heterozygous in one *NACβ* gene in the mutant background of the other one (*nacβ1/+;nacβ2*, and *nacβ1;nacβ2/+*, respectively) were let to self-pollinate. Their offspring was cultivated and conventionally genotyped by PCR with a set of insert-specific and gene-specific primer pairs (Table 1). The number of heterozygous plants (*nacβ1/+;nacβ2* or *nacβ1;nacβ2/+*, respectively) was nearly the same in both self-crosses as expected by the Mendelian laws of inheritance (13% more plants in *NACβ1*, and 10% more in *NACβ2*). On the contrary, the number of homozygous wild type plants (*NACβ1nacβ2* or *nacβ1NACβ2*, respectively) was in both cases higher than expected (11% more in *NACβ1*, and 29% more in *NACβ2*), whereas the number of homozygous mutants (*nacβ1nacβ2* or *nacβ1nacβ2*, respectively) was lower than the Mendelian one (46% less in *NACβ1*, and 48% less in *NACβ2*). Based on these self-crosses, it was deduced that the mutant allele of both studied genes showed a reduced transmission to the next generation compared to the wild type allele.

To further elucidate the transmission efficiency of the mutant alleles via male and female gametophyte, two rounds of reciprocal out-crosses were carried out, each of which was represented by four crosses, i.e., eight crosses in total. The first round studied the transmission efficiency of one *NACβ* gene on the wild type background of the other *NACβ* gene, whereas the second round considered the transmission efficiency of one *NACβ* gene on the mutant background of the other *NACβ* gene. (i) In the first round, the heterozygous plants in one *NACβ* gene on the mutant background of the other one (*nacβ1/+;nacβ2*, and *nacβ1;nacβ2/+*, respectively) were crossed with Col-0 wt plants (genotype *NACβ1NACβ2* in both cases). These crosses were performed in two ways, the first time the pistils of *nacβ1/+;nacβ2*, or *nacβ1;nacβ2/+*, respectively, were pollinated with wild type pollen, whereas the other time their pollen was used for the pollination of wild type pistils. Such setup of crosses enabled to test the transmission efficiency of the mutant allele via both female and male gametophytes (Table 2). The transmission efficiency of the mutant and the wild type allele was calculated from the occurrence of homozygous mutant and heterozygous plants in the offspring of these crosses. Theoretically, they should occur with the same frequency. The *nacβ1* mutant allele showed higher transmission efficiency compared to the wild type allele via both female (+20%), and male gametophyte (+52%). On the contrary, the *nacβ2* mutant allele was transmitted with a slightly reduced efficiency than wild type alleles were, again both via female (−15%) and male gametophyte (−22%). (ii) The second round of out-crosses was performed on the mutant background of the non-tested *NACβ* gene. The tested gene was heterozygous in one crossed plant (*nacβ1/+;nacβ2*, and *nacβ1;nacβ2/+*, respectively) whereas in the second plant was represented by two wild type alleles (*NACβ1nacβ2*, and *nacβ1NACβ2*, respectively). Again, two crosses were performed for each *NACβ* locus, once looking for transmission efficiency via male and the second time via female gametophyte (Table 3). The mutant allele of *NACβ1* showed a comparable transmission efficiency as the wild type allele did both via female (−5%) and male gametophyte (−1%). Similarly, the mutant allele of *NACβ2* was transmitted with a slightly worse efficiency than the wild type alleles were both via female and male gametophyte (both times 17% less). To sum up, the *nacβ1* mutant allele showed a comparable male and female transmission efficiency on the mutant background of the other *NACβ* gene, and even better male and female transmission efficiency on the wild type background. On the contrary, the *nacβ2* mutant alleles showed slightly less efficient transmission by both male and female gametophyte on both backgrounds of the other *NACβ* gene (mutant and wild type).

Taken together, the self-crosses and out-crosses showed a reduced transmission efficiency of *nacβ2* mutant allele compared to the wild type regardless on the background of the *NACβ1*. On the other hand, the *nacβ1* mutant allele showed a reduced transmission efficiency in the self-cross, a comparable transmission efficiency in the outcrosses on the mutant background of the *NACβ2* gene whereas its transmission efficiency was even higher in the outcrosses on the wild type background of *NACβ2*.

### 2.4. The *nacβ1nacβ2* Mutant Phenotype Was Complemented by any Functional NACβ Allele

After testing the transmission efficiency of the mutant alleles, it was necessary to prove that the *nacβ1nacβ2* phenotype was caused by the insertions in both *NACβ*-coding genes. The first experiment tested whether the mutant phenotype would be reverted after a new functional copy of a single *NACβ* gene (containing native promoter and coding genomic sequence tagged with GFP) was inserted to the *nacβ1nacβ2* plants. However, due to the reduced seed set of the *nacβ1nacβ2* plants, we were not able to recover enough transformants, so the experiment was repeated by transforming plants having one original functional copy of one *NACβ* gene, i.e., *nacβ1/+;nacβ2*, and *nacβ1;nacβ2/+*. Every time, the construct carried the same *NACβ* gene, alleles of which were represented as homozygous mutant in the transformed plants, so *nacβ1;nacβ2/+* plants were transformed with proNACβ1::NACβ1–GFP construct, whereas the *nacβ1/+;nacβ2* plants were transformed with proNACβ2::NACβ2–GFP construct. Several *nacβ1nacβ2* plants carrying the construct (either with NACβ1, or NACβ2) segregated in the next generation, and one novel functional allele of any *NACβ* gene rescued the phenotypic defects of the *nacβ1nacβ2* mutants, which became undistinguishable from the Col-0 wt plants (Figure 1I–L).

In parallel, *nacβ1nacβ2* plants were crossed with Col-0 wt, again reciprocally. The resulting heterozygous plants in both *NACβ* loci (*nacβ1/+;nacβ2/+*) did not show the mutant phenotype regardless of the cross direction and looked like the Col-0 wt plants. The complementation analysis together with the backcross verified that the observed *nacβ1nacβ2* phenotype was caused by the insertions in both *NACβ* loci and that its phenotype can be rescued by presence of any single functional *NACβ* allele.

Then, the opposite effect of the NACβ expression was studied, the effect of overexpression. Each *NACβ* gene represented by its genomic coding sequence was expressed under the 35S cauliflower mosaic virus (CaMV) promoter, which shows a strong expression in most *A. thaliana* sporophyte tissues [38]. The 35S-driven overexpression of each NACβ separately as well as both at the same time did not lead to any phenotypic changes and the transformed plants looked like the conventional Col-0 wt. Then the overexpression was also performed in male gametophyte (under the LAT52 promoter [39]) and female gametophyte (under DD33 promoter [40]). Again, no phenotypic defects were observed.

### 2.5. The NACβ Proteins Localized into Cytoplasm and Nuclei and Their Promoters were Active in Multiple Organs

**Figure 3:**
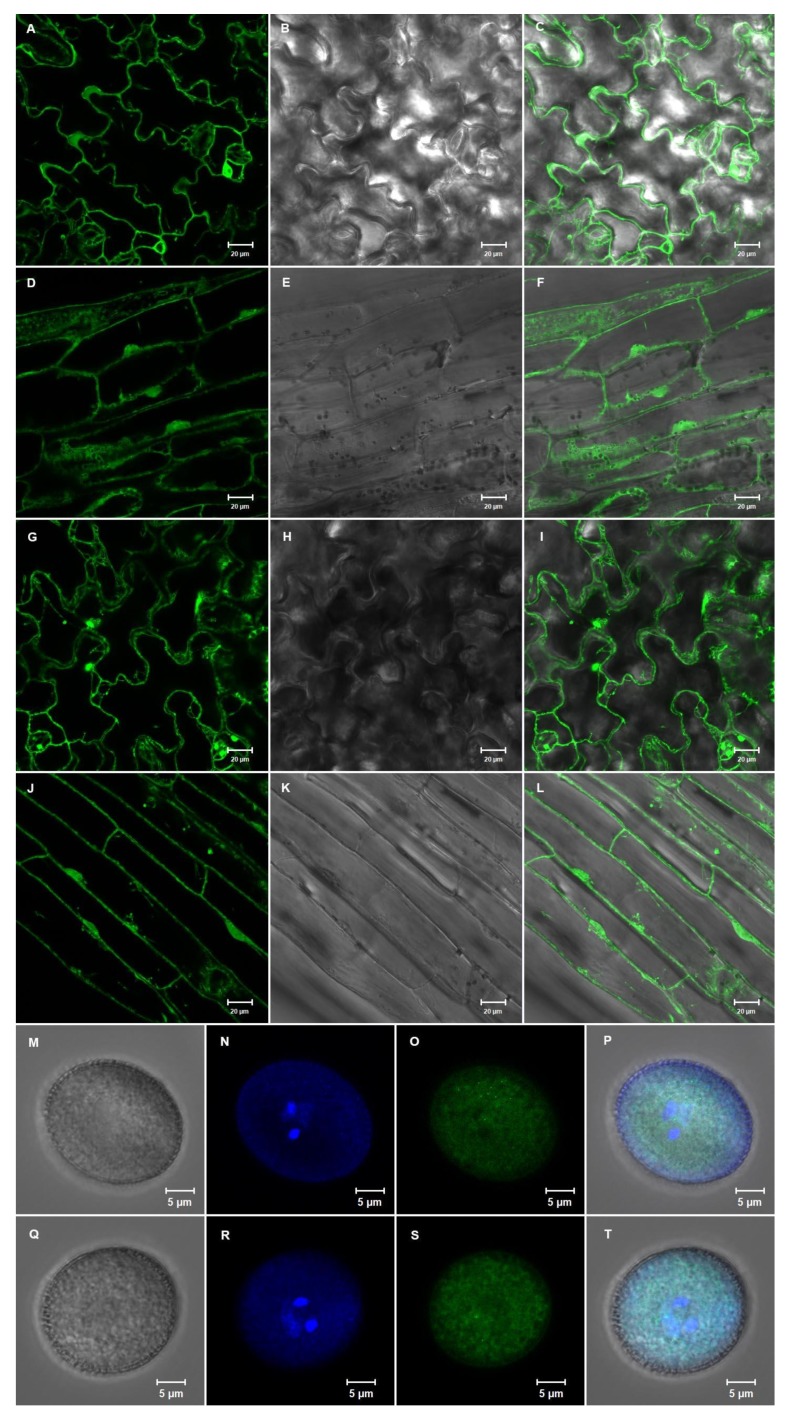
The subcellular localization of NACβ1 and NACβ2 expressed in ten-day seedlings under their own promoters, and the expression of NACβ1 and NACβ2 under the LAT52 promoter in vegetative cells of the pollen grains observed under the confocal microscope. (**A**–**F**)—proNACβ1::NACβ1–GFP, scale bars represent 20 µm. A–C—Leaves. D–F—Hypocotyl. (**G**–**L**)—proNACβ2::NACβ2–GFP, scale bars represent 20 µm. G–I—Leaves. J–L—Hypocotyl. (**M**–**P**)—proLAT52::NACβ1–GFP in vegetative cells of the pollen grains, scale bars represent 5 µm. (**Q**–**T**)—proLAT52::NACβ2–GFP in vegetative cells of the pollen grains, scale bars represent 5 µm. (**A**,**D**,**G**,**J**,**O**,**S**)—GFP fluorescence. (**B**,**E**,**H**,**K**,**M**,**Q**)—Bright field. (**N**,**R**)—DAPI staining. (**C**,**F**,**I**,**L**,**P**,**T**)—All signals merged together.

**Figure 4:**
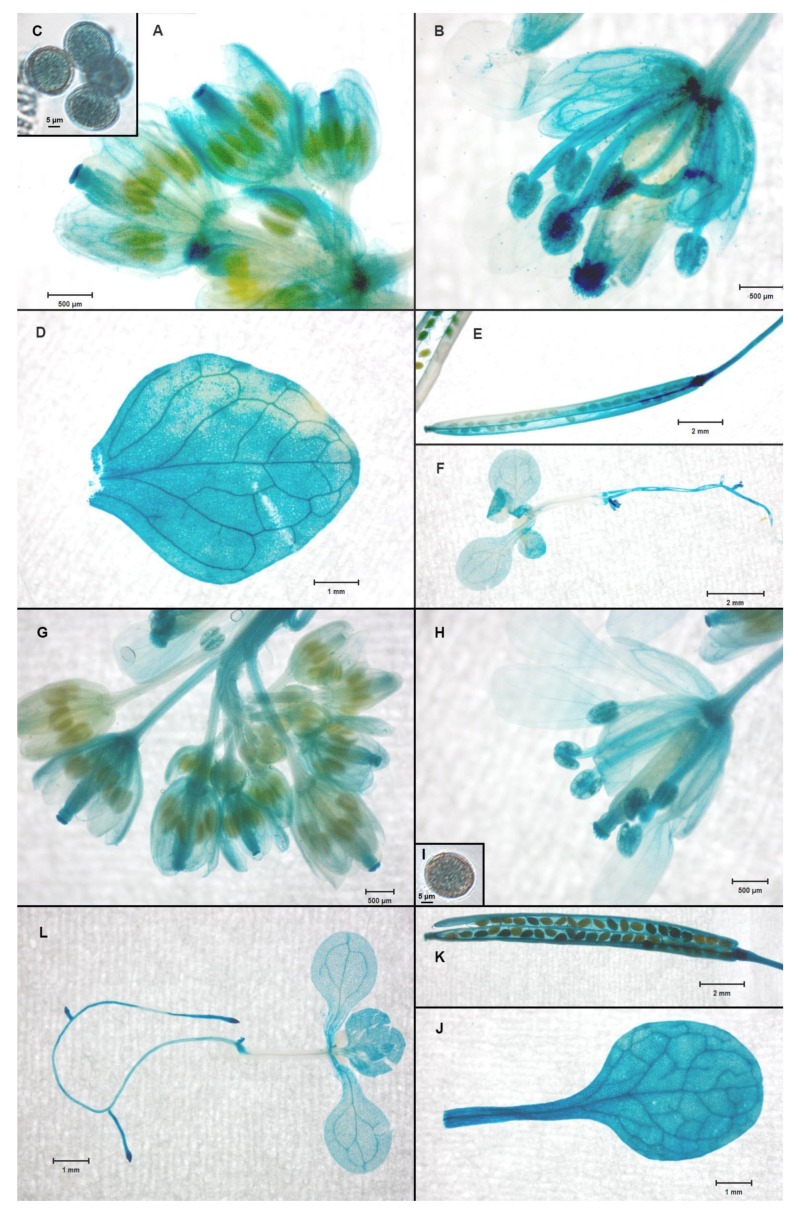
The promoter analysis of NACβ1 and NACβ2 fused with glucuronidase, observed under the dissection microscope (all images except for (**C**), and (**I**)) and optical microscope (**C**,**I**). The construct carried NACβ1–GUS (**A**–**F**) or NACβ2–GUS (**G**–**L**). (**A**,**G**)—Inflorescence. (**B**,**H**)—Flower. (**C**,**I**)—Pollen grain. (**D**,**J**)—Leaf. (**E**,**K**)—Silique. (**F**,**L**)—Ten-day old seedling. Scale bars represent 5 µm (**C**,**I**), 500 µm (**A**,**B**,**G**,**H**), 1000 µm (**D**,**J**,**L**), or 2000 µm (**E**,**F**,**K**), respectively.

To determine the subcellular localization, the same constructs were used as for the complementation (proNACβ1::NACβ1–GFP, and proNACβ2::NACβ2–GFP). The 10-day *A. thaliana* seedlings, which were transformed by *Agrobacterium tumefaciens*, were selected on MS/2 plates with proper antibiotics. The acquired selectants were observed under the confocal microscope. The genes coding for both *NACβ* genes (*NACβ1*, and *NACβ2*) showed the same subcellular localization, since they both were localized inside the cells of the hypocotyls and the leaves to cytoplasm, and the nuclei (Figure 3A–L). In mature pollen, NACβ1 and NACβ2 proteins were observed inside granular structures in the vegetative cell cytoplasm but not inside the vegetative nuclei (Figure 3M–T). Then, the promoter analysis was performed by glucuronidase marker gene driven directly by each *NACβ* promoter (proNACβ1–GUS, and proNACβ2–GUS). The 10-day old seedlings and several organs (leaves, cauline leaves, flowers, pollen grains, and siliques together with seeds) from older plants were stained for the presence of glucuronidase activity and observed under dissection microscope. Both *NACβ* genes showed very similar pattern of promoter activity (Figure 4), so none of them showed any organ specificity compared to the other gene, and so was in accordance with the published transcriptomics data [41,42]. Both genes were active in the whole seedlings except for their hypocotyls, and then in leaves, cauline leaves, flower buds, flowers, pollen grains, siliques, and seeds. Inside flowers, their activity was most prevalent in pistils, and anthers. Taken together, the genes showed a house-keeping nature of their promoters since they were present nearly in the whole plant.

### 2.6. RNA Sequencing of the *nacβ1nacβ2* Flower Bud Transcriptome Revealed Genes Important for Stress Responses and Male Gametophyte Development

Since the phenotypic defects of the *nacβ1nacβ2* plants were most prevalent in the flowers and siliques, and the data from pollen cultivation in vitro, pollen cultivation *in vivo* and blue dot assay showed that the defects are likely in both gametophytes and/or sporophyte, flower buds (including the stages up to the stage 12 [43]) were selected as the studied organ to search for genes, transcription of which was influenced in the *nacβ1nacβ2* plants. To accomplish this task, two approaches were chosen, transcriptomics and proteomics. Moreover, to see whether the single phenotype-less *nacβ* mutants (*nacβ1* or *nacβ2*) bear any changes on the transcriptomic level, they were also included in the analysis. RNA genome-wide transcriptional analyses were performed on Illumina platform and yielded approximately 40.6 million paired-end 101 base pair (bp) reads per sample after quality (>Q20) control and technical sequences trimming. On average 94.3% of reads were uniquely mapped to *A. thaliana* reference genome (TAIR10). 

**Figure 5:**
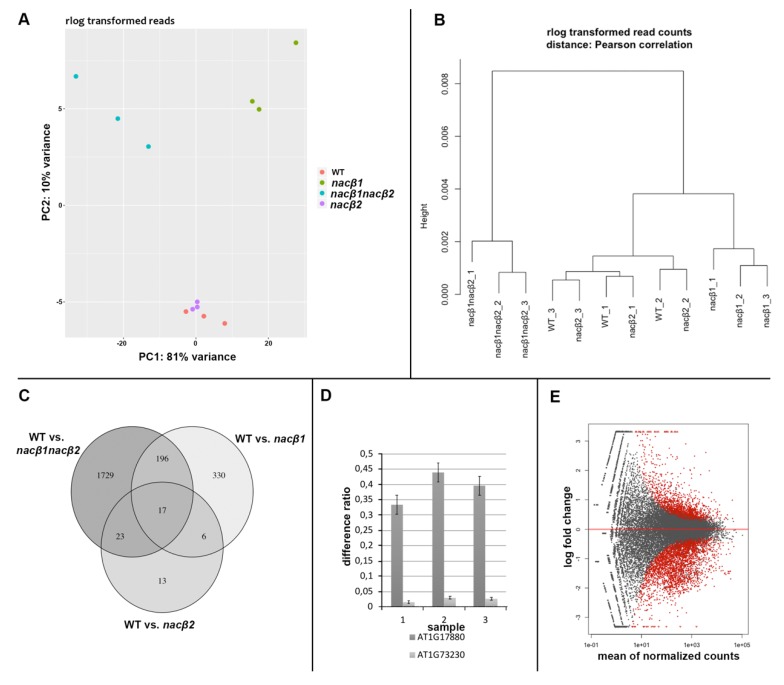
Analyses of flower bud transcriptome. (**A**)—PCA based clustering of regularized log transformed read counts of four analyzed genotypes *nacβ1nacβ2, nacβ1, nacβ2,* and Col-0 wt explaining 81% of PC1 variance and 10% of PCA2 variance shows three distinctive clusters. (**B**)—Hierarchical clustering of regulated log transformed read counts of four genotypes *nacβ1nacβ2, nacβ1, nacβ2* and Col-0 wt based on Pearson correlation distance shows three distinctive clusters. (**C**)—Venn diagram showing comparison of DEG numbers in *nacβ1nacβ2, nacβ1, nacβ2* and Col-0 wt. (**D**)—Relative expression of *NACβ1* (At1g73230) and *NACβ2* (At1g17880) in *nacβ1nacβ2* when compared to expression in Col-0 wt showing knock-out of NACβ1 and knock-down of NACβ2. (**E**)—MA plot of gene expression in *nacβ1nacβ2* compared to Col-0 wt. Genes with adjusted *p*-value < 0,05 are shown in red.

**Figure 6:**
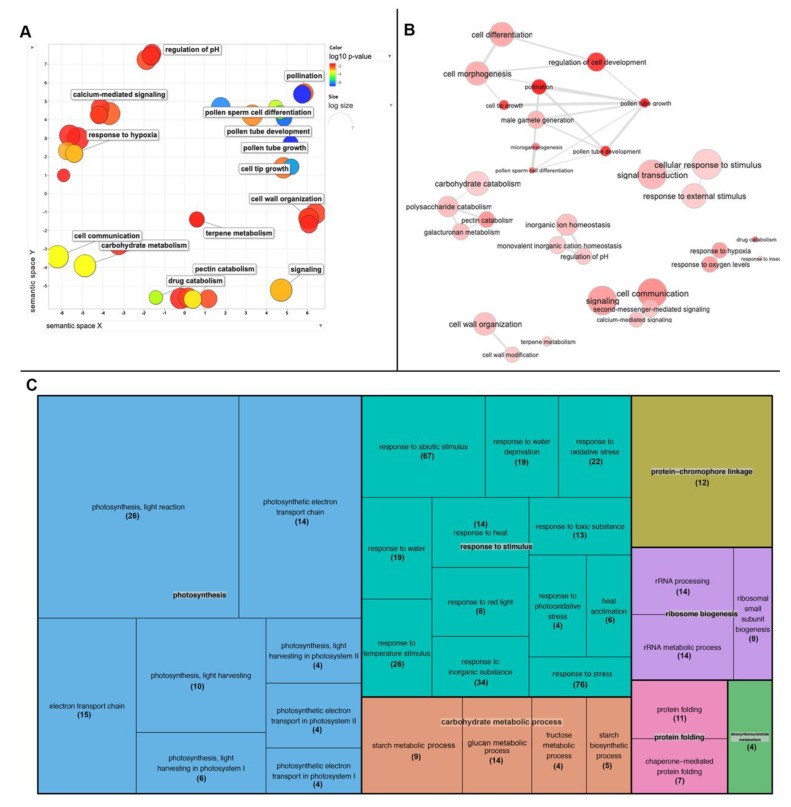
Functional analysis of DEGs and DEPs in *nacβ1nacβ2.* (**A**)—Enrichment analyses of gene ontology (GO) biological process scatter plot for 1909 DEGs. Names of representative GO terms that were present in the clusters are shown. The bubbles without any name represent other relative GO terms. The various colors indicate log_10_ FDR value of GO enrichment analysis for respective GO term, whereas the distance indicates semantic similarity of the categories. Bigger size of bubbles indicates more parent GO terms. (**B**)—Interactive graph of GO biological process terms of 1909 DEGs. GO terms are connected according to their relationship. Bigger size of bubbles indicates more parent GO terms. Brighter color indicates lower log_10_ FDR value of GO enrichment analysis for respective GO term. (**C**)—Enrichment analyses GO biological process tree map constructed with REVIGO for 399 differentially expressed proteins (DEPs). Each square represents a GO term. The same color connects GO terms that belong to the same parent category. The name of parent category is highlighted in bold. The size of squares is proportional to the FDR value for that term enrichment analysis. One gene can be present in more categories. The maps were constructed with ReviGO [44].

Of these, differentially expressed genes (DEGs) with adjusted p-value < 0,05, and FoldChange ≥±2 were selected. Principal component analyses (PCA) with PC1 explaining 81% variance and PC2 10% as well as in Pearson correlation hierarchical clustering analysis, three clusters are revealed: *nacβ1nacβ2*, *nacβ1*, and *nacβ2* together with Col-0 wt (Figure 5A,B). The clustering corresponded to the observed phenotype and to the identified DEGs. There were identified in total 1965 DEGs in *nacβ1nacβ2* (Table S3), 59 DEGs in *nacβ2*, and 549 DEGs in *nacβ1* (Figure 5C). Moreover, only *NACβ1* was differentially expressed in the *nacβ1nacβ2* mutant leading to nearly total silencing but on the contrary, the expression of *NACβ2* was only lowered (Figure 5D). Out of the total 1965 DEGs in the *nacβ1nacβ2*, 363 were up-regulated and 1602 down-regulated (Figure 5E). To further analyze functional potential of the DEG list, the transcripts were classified according to biological processes, molecular function and cellular component using gene ontology (GO) enrichment analysis. There were successfully recognized 1919 DEGs by the software, which were used for the enrichment analyses. Of these, 91 (87 downregulated) DE male germ-line connected genes were included in five enriched biological processes GO terms, namely “pollen development”, “pollen sperm cell differentiation”, “pollen tube development”, “pollen tube growth”, and “regulation of pollen tube growth” (Table S3). 19 of these DEGs with 14 others were also included in the enriched “pollen tube” GO cellular component category. “Pectin catabolic process” and “cell wall modification” enriched GO biological process GO terms included 32 DEGs with 8 pectinesterases, 10 pectinesterase inhibitors and 6 pectine lyases. “Cell wall” was enriched in cellular component and “pectinase activity” in molecular function. “Protein complex oligomerization” enriched GO term included 12 small heat shock family genes. In the DEGs list, there were upregulated 18 genes encoding other heat shock proteins and 2 genes encoding heat shock factors. Among other enriched biological process GO terms, there were “regulation of pH” with 10 DE Cation/H(+) antiporters, “calcium-mediated signaling”, “terpene metabolism”, “response to hydrogen peroxide”, and “response to insect”. Visualization of GO enrichment analysis is showed in Figure 6A,B. The transcriptomics data were validated by measuring expression of 12 candidate genes by quantitative qRT-PCR. The quantities of these genes according to qRT-PCR corresponded to expression values obtained from next generation sequencing with correlation coefficient R^2^ = 0,7299 (Figure S3).

### 2.7. Proteomic Analysis of the *nacβ1nacβ2* Flower Buds Revealed Proteins with Role in Photosynthesis and Stress Responses

To acquire a closer insight into the regulatory networks influenced by the knock-down of both NACβ subunits, the proteomic analysis of the *nacβ1nacβ2* flower buds was performed and compared to the Col-0 wt plants. In total, there were 5499 protein groups reported that were identified according to at least two peptides in at least two out of three biological replicates in at least one of the samples and have measured intensity in at least two replicates in at least one sample. For the differentially expressed proteins, the following criteria were set: the fold change between the *nacβ1nacβ2* and the wt had to be ≥2 and the adjusted p-value < 0.05. From the total 5499 analyzed protein groups, 460 were differentially expressed in *nacβ1nacβ2,* with 290 downregulated and 170 upregulated ones (Table S4). All proteins were mapped with GO enrichment analyses. 38 differentially expressed proteins (DEPs) connected to photosynthesis were downregulated in the *nacβ1nacβ2.* They were present in the enriched GO terms “photosynthesis”, “response to red light”, “photosystem electron transport chain in photosystem I”, “protein-chromophore linkage”, “photosynthesis, light harvesting in photosystem I”, and “photosynthesis, light harvesting in photosystem II” of biological processes. In cellular component category, chloroplast localization was represented by “chloroplast envelope”, “chloroplast thylakoid lumen”, “plastoglobuli”, and “photosystem I reaction center”. Biological process-enriched GO terms connected to stress response were represented by “water deficient”, “response to toxic substances”, “response to oxidative stress”, and “response to heat”; they included 44 primary downregulated proteins. “Response to heat” was represented by 7 upregulated chaperones which were also present in “protein-folding” enriched GO term together with 5 other proteins. 6 of these chaperones were connected directly to de-novo folding. Other interesting GO terms were represented by “rRNA processing”, “ribosome biogenesis”, and “ribosome small subunit assembly” and metabolism terms “starch metabolism”, and hexose metabolism”. Visualization of GO enrichment analysis is showed in Figure 6C.

### 2.8. The Germination Efficiency of *nacβ1nacβ2* Seeds Was Lower Under the Salt and Osmotic Stresses

Since the transcriptomic and proteomic analyses revealed several regulated genes connected with stress responses (e.g., chaperones), sensitivity of *nacβ1nacβ2* seed germination to salt and osmotic stress compared to the Col-0 wt was tested. The sodium chloride (NaCl) delayed the germination process and reduced the number of germinated *nacβ1nacβ2* mutant seeds already at 50 mM concentration (Figure S4A). The *nacβ1nacβ2* germination was more influenced by 100 mM and 125 mM NaCl since the total number of germinated seeds gradually decreased with an increasing NaCl concentration. These concentrations (50 mM, 100 mM, and 125 mM) affected the Col-0 wt seeds rather by delaying germination since their germination rate remained nearly unaffected after the last scoring in 13 days (Figure S4C,E). On the contrary, 150 mM NaCl significantly reduced the total number of germinated seeds of both *nacβ1nacβ2* and wt (Figure S4D), representing probably a too high concentration of NaCl for the seeds to survive. 

The influence of mannitol on the germination of *nacβ1nacβ2* seeds was similar to the influence of NaCl. Under 250 mM and 300 mM concentrations, the *nacβ1nacβ2* germination was delayed and the number of germinated seeds was slightly reduced, whereas Col-0 wt seeds were rather affected by a slightly delayed germination (Figure S4B). On the other hand, the influence of 350 mM mannitol was comparable to the effect of 150 mM NaCl since the germination of both *nacβ1nacβ2* and wt was significantly slowed down and the number of germinated seeds reached only around 60% of the control if observed 13 days after seed sowing.

## 3. Discussion

### 3.1. Phenotype Analysis of *nacβ1nacβ2*

The *nacβ1nacβ2* mutant showed a notable phenotype: delayed development, lower amount of chlorophyll in the leaves, unusual number of flower organs, and shorter siliques with a reduced seed set. Similarly, in *Nicotiana benthamiana* [24] and wheat (*Triticum aestivum* [25]), the NACβ downregulation caused an abnormal leaf morphology together with a lower chlorophyll content (and thus leaf yellowing) whereas the size of the whole plant remained the same. Our experiments testing the behavior of the male and female gametophytes revealed that the *nacβ1nacβ2* plants showed likely defects in sporophyte tissues of the flowers and/or defects in both gametophytes as the germination rate of pollen tubes was reduced together with the velocity of the growth since they reached shorter distance in the same time as the wild type did. Furthermore, the targeting efficiency was diminished also on the female side since wild type pollen tubes were less efficiently attracted by the *nacβ1nacβ2* ovules. The targeting efficiency was the worst in case the *nacβ1nacβ2* ovules were targeted by *nacβ1nacβ2* pollen tubes. 

The *NACβ* genes are both quite strongly expressed according to the transcriptomic datasets presented in eFP browser [45], and according to the strong GUS expression driven by NACβ promoters in nearly all organs throughout plant development (Figure 4). Although the observed phenotype was quite clear, one would probably expect a more severe phenotype if the mutants represent a knock-out/down of such strongly expressed genes with a “housekeeping” function in translation. Moreover, in several model animals (for instance mouse, nematodes and drosophila), the NACβ knock out caused embryonic lethality [14,15,16]. Thus, it is likely that the NACβ function is mimicked in case of its knock-out to some extent by other chaperones since their transcripts were more abundant in the *nacβ1nacβ2* transcriptome compared to the wild type (Table S3). This hypothesis was further supported by yeast experiments, in which the loss of the whole NAC complex did not affect the yeast growth under the optimal conditions [4] whereas the yeast cells with a knocked out NAC complex together with two heat shock proteins 70 (Hsp70) chaperones SSB (stress 70 B) were still viable but their growth was notably slowed down [13]. Thus, Hsp70 chaperones in yeast can to some extent complement NAC’s function. It should be also mentioned that the survival and limited fertility of the studied *nacβ1nacβ2* plants were likely enabled by the fact that one *NACβ* gene (*NACβ2*) was knocked down rather than knocked out, and the remnants of its transcript could carry some basic function in the *nacβ1nacβ2* plants. On the contrary, the second *NACβ* gene (*NACβ1*) was proven to be knocked out completely. Last but not least, it is worth mentioning that the knock out of some house-keeping genes showed rather a tissue-specific phenotype than a severe phenotype affecting dramatically the whole plant [46]. The different nature of both alleles could also explain the different transmission efficiency of the *NACβ1* and NACβ2 mutant alleles since the knocked-down allele likely still carries out its regulatory function. Thus, the knock-down allele of *NACβ2* seemed to be more harmful than the knock-out allele of *NACβ1*. The higher transmission efficiency of the *NACβ1* mutant allele than the one of the wild type allele was likely caused by the wild type background of the *NACβ2* gene, which could carry out the *NACβ1* function. Moreover, it is likely that both *NACβ* genes are likely regulated together.

The flower phenotype tempted us to speculate whether meristem organizing, or flower development genes had a changed abundance in the *nacβ1nacβ2* transcriptome compared to the Col-0 wt plants. Meristem development disruption can be exhibited in several aspects, one is organ identity aberration, which was disrupted for example in *apetala 3 (ap3), pistillata (pi),* or *agamous (ag)* mutants [47], whereas the *clavata3 (clv3)*, *clavata1 (clv1)*, and *wiggum (wig)* mutants possessed a changed meristem size and, consequently, aberrations in organ numbers [48]. Another option can be connected to the duration of meristematic maturation. Prolonged meristematic activity under certain conditions led to larger meristem and then also to changes in organ number. This phenomenon was recently observed in close *A. thaliana* relative *Cardamine hirsuta* [49]. However, despite the observed phenotype, the abundance of the meristem organizing, or flower development genes was not significantly changed in the *nacβ1nacβ2* flower bud transcriptome. Thus, we speculate that the stress-like conditions that were shown by the transcriptomics data, where many heat shock proteins were upregulated in the *nacβ1nacβ2*, could lead to a prolonged meristematic activity and thus to disrupted number of floral organs and in general could alter protein homeostasis.

### 3.2. NACβ Paralogues

Interestingly, the above-mentioned phenotype was observed exclusively in the *nacβ1nacβ2* plants since the presence of any single functional *NACβ* allele (regardless whether *NACβ1* or *NACβ2*) suppressed the mutant phenotype. Such an observation was done independently three times, first when crossing the homozygous plants of two T-DNA insertion lines (SALK_043673 bearing insert in *NACβ1*, and GK368-H02 with insert in *NACβ2*), the phenotype did not appear until double homozygous mutants (*nacβ1nacβ2*) were acquired, so plants with one functional allele (either *nacβ1/+;nacβ2*, or *nacβ1;nacβ2/+*) did not show any phenotypic changes. Second, the *nacβ1nacβ2* plants (with the above phenotype) were backcrossed with Col-0 wt (genotype *NACβ1NACβ2*), giving rise to double heterozygous plants (*nacβ1/+;nacβ2/+*), again with no phenotypic defects. Third, the *nacβ1nacβ2* phenotype was reverted to the wild type phenotype by creating the transgenic plants carrying one functional copy of any *NACβ* gene. 

Similar behavior to the alleles of the *NACβ* genes was observed for instance in case of *SEPALLATA* (*SEP*) since only its triple mutant *sep1/sep2/sep3* showed a severe flower phenotype as its flowers carried sepal-like structures instead of the proper flower organs, whereas in contrast, any of its single mutants showed only a subtle phenotype [50]. Such a behavior was explained by the redundancy of these three *SEPALLATA* paralogues. In case of *NACβ*, the phenotype was suppressed, and the function was restored by single functional copy of any *NACβ* genes, indicating that both genes were functionally redundant and very similar to each other. Due to the high level of sequence similarity between these two proteins (reaching 89% similarity on the amino acid level where most differences were in the C-terminal part of the protein), it is likely that both *NACβ* genes show a similar function. Furthermore, both *NACβ* genes showed a similar subcellular localization together with promoter activity and none showed any spatial or temporal specificity. Unlike two yeast *NACβ* genes, which showed a distinct function and a different efficiency for creating the dimer complexes with the single yeast *NACα* gene [3], *A. thaliana NACβ* genes seem to be much closer to each other and rather share the same function. However, in the future on-going experiments, it remains to be tested which of the *NACα*, and *NACβ* homologues appear in the NAC dimers and if there are any binding preferences between them at all. 

The β-subunit was first reported as a part of NAC heterodimer together with α-subunit [7] but both subunits were also reported to play different roles outside the heterodimer by influencing gene expression [18,51,52]. The mutual regulation of α- and β-subunits was also proposed, claiming that NACα transcriptional coactivator function is inhibited by the presence of NACβ to promote the heterodimer formation [53]. Nevertheless, the RNAseq data from *nacβ1nacβ2* flower buds did not show any changes in the abundance of NACα subunits in the *nacβ1nacβ2* plants compared to the wt, therefore it can be concluded that on the transcriptomic level in the flower buds, NACβ did not influence the expression of NACα. On the other hand, on the proteomic level, there was shown the downregulation of NACα3 (At5g13850), NACα4 (At4g10480), and NACα5 (At1g33040) in *nacβ1nacβ2* making likely the expression regulation on the level of translation. 

### 3.3. Subcellular Localization of NACβ Proteins

Both paralogues encoding NACβ proteins were localized into both cytoplasm and nucleus in *Arabidopsis thaliana* seedlings (Figure 3A–L) regardless of whether N-terminal or C-terminal GFP fusion was employed. There were not any differences in the localization of both homologues, thus the localization also supported the likelihood of functional similarity of these two *NACβ* genes. Our localization data represented by in frame fusion of NACβ coding sequences with green fluorescent protein, were consistent with the subcellular localization of both *Arabidopsis thaliana* NACβs [21], and wheat (*Triticum aestivum)* NACβ localization in *A. thaliana* protoplasts [25]. The protein localization is also in accordance with several software predictions, where the NACβ proteins were predicted to be localized either to nucleus or cytoplasm. For instance, NucPred [54] predicted NLS in both NACβs, represented in both proteins as amino acids 22-25 (RRKK) with the score 0.40 in NACβ1 or 0.44 in NACβ2, respectively. However, these NLS predictions were not strong, which agreed with the dual targeting (nucleus, and cytoplasm). This is also in accordance with the cytoplasmic localization of both NACβs in the vegetative cell of the pollen grain (Figure 3M–T), the NLS being likely too weak for these tissues. The combined localization also accords with the NAC function, either as a heterodimeric complex binding the nascent polypeptide or each subunit individually as a transcription factor or activator, the former localized likely to cytoplasm, whereas the latter function requires nuclear localization, reviewed by Kogan and Gvozdev (2014), and Rospert et al. (2002) [1,3]. In the cytoplasm, the NACβ1 (At1g73230) was reported to bind to the ribosomes, which was mediated by phosphorylation carried out by OST1 protein [21]. 

### 3.4. Flower Bud Transcriptome and Proteome

The presented transcriptomic and proteomic analyses showed the genes that were influenced in their abundance in the floral buds of *nacβ1nacβ2* mutants compared to the Col-0 (wt) plants. Next generation sequencing of the flower bud transcriptome followed by differential expression (DE) analysis showed 1965 differentially expressed genes (DEGs) between the *nacβ1nacβ2* mutant and Col-0 wt plants, whereas there were only 59 and 549 DEGs in single *NACβ* mutants *nacβ2* and *nacβ1,* respectively. These results were in accordance with the observed phenotype since *nacβ1nacβ2* mutants showed a notable phenotype (delayed development, lower amount of chlorophyll in leaves, flowers with a different number of flower organs, shorter siliques, reduced seed set) whereas both single mutants were undistinguishable from the Col-0 wt plants (see in more detail above). Thus, it was unlikely that plants of the same look as the wild types will have many transcripts of a different abundance.

From GO term enrichment analysis, three groups of genes emerged. Firstly, there were 87 downregulated genes connected to male germ-line development from sperm-cell differentiation to pollen tube development and growth. Lower germination rate, slower growth and lower fertilization rate were also observed in pollen tubes of the *nacβ1nacβ2* plants. The presence of transcripts from different pollen developmental stages was probably caused by the nature of collected flower bud tissue which included flowers up to stage 12 [43]. From the stage 10 do 12, pollen mitosis I and II take place, so there were likely present both bicellular and tricellular pollen in the acquired samples. Also, as described in other plant species, mRNAs in developing pollen are stored in translationally inactive complexes [55,56]. Pollen activation is then followed by massive translation of these pre-prepared mRNAs, enabling, among others, rapid growth of pollen tube [57]. This could explain high amount of pollen tube regulatory genes in the analyzed RNA. The list consisted of genes involved in various molecular processes, many of which were connected to reproduction defects upon silencing. Among these playing role in pollen tube development and growth, there were heterodimeric MADS box agamous-like proteins AGL66 (At1g77980) and AGL104 (At1g22130). Double mutant *agl66 agl104* exhibits delayed pollen grain germination, aberrant pollen tube growth, reduced pollen viability and fertility [58]. Also, RALF4 (At1g28270) and RALF19 (At2g3375), peptide ligands responsible for pollen tube integrity, were present [59]. Four pectin catabolism genes, two pectin esterase inhibitors PMEI1 (At1g48020) and PMEI2 (At3g17220) and two pectinesterases PME1 (At1g69940) and PME5 (At1g10770) were downregulated. Cell wall modification proteins are especially important for pollen tube cell wall rigidity and dynamics [60]. In sperm-cell development category, sperm cell specific MYB transcription factor DUO1 (At3g60460) and 13 genes encoding DUO1 targets were downregulated. DUO1 is responsible for division of germ cell into two sperms cells via regulation of mitotic cyclin CYCB1 accumulation during G2 phase [61]. Among its targets, there was downregulated DMP9/DAU2 (At5g39650), which is responsible for double fertilization and its silencing leads to seed abortion [62]. Last but not least, three zinc finger transcription factors DAZ1 (At2g17180), DAZ2 (At4g35280), DAZ3 (At4g35700) or vacuolar aquaporin TIP5;1 (At3g35700) important for pollen tube growth were included. In summary, these 87 downregulated genes could be collectively responsible for the observed pollen phenotype. 

The second group consisted of 18 upregulated heat shock proteins. There were three HSP70 family proteins HSP70-4 (AT3G12580), BiP3 (HSP70-8, AT2G32120), and HSP70-T (AT3G32120) together with HSP90.1 (At5g52640), and HSP101 (At1g74310). HSP70 forms a heterodimer with HSP40 to solubilize protein aggregates and has its role in stress tolerance. BiP3 together with BiP1 and BiP2 were expressed exclusively under stress conditions. Decrease in activity of these chaperones led to a worse pollen tube growth [63]. There were also 3 small heat shock proteins (HSP18.2 AT5G59720, HSP17.4 AT3G46230, and HSP17.6A AT5G12030) and 9 HSP20-like family proteins (AT1G53540, ATHSP22.0 AT4G10250, AT2G29500, AT5G51440, AT1G07400, AT1G59860, AT5G37670, AT1G54050, and AT4G21870), which play their role in both solubilizing protein aggregates, and in plant heat stress and immunity. They are usually less abundant and with a spatially- or temporally-specific expression throughout plant development, such as embryonic development and seed maturation [64,65]. In the DEG list, there were also 18 downregulated LEA proteins, which act as chaperones during water deficiency and as such promote dehydration stress tolerance. 

In the GO enrichment analyses of the proteome, 38 photosynthetic downregulated proteins were identified in the *nacβ1nacβ2* flower buds. These proteins were mainly connected to light harvesting on photosystem I or II, response to red light or electron transport chain. There were 6 chlorophyll a-b binding proteins (LHCB2.2 AT2G05070, LHCB3 AT5G54270, LHCB4.1 AT5G01530, LHCB4.2 AT3G08940, and lhb1B2 AT2G34420). They represent light harvesting complexes in the thylakoid membrane. Silencing of LHCB led to the modulation of ROS levels and decrease in sensibility to ABA during stomatal opening, which consequently led to lower stress tolerance [66]. Moreover, ABA1 (AT5G67030), zeaxanthin epoxidase responsible for the first step of ABA biosynthesis, was downregulated. Seven other proteins were parts of photosystem I (LHCA6 AT1G19150, psaC ATCG01060, psaA ATCG00350, psaD1 AT4G02770, psaE1 AT4G28750, psaE2 AT2G20260, and psaN AT5G64040). psaE1 mutants exhibit pale green leaves [67]. Also, photosystem repair and stress tolerance proteins were present as well as protein D1 (ATCG00020) and protein D2 (ATCG00270) of photosystem II. Taken together, downregulation of photosynthetic proteins supports lower chlorophyll content phenotype observed in the mutant. It may also lead to the delayed development of *nacβ1nacβ2* plants. Besides, it may suggest that beside ER and potential mitochondria targeting of nascent polypeptide chains, NAC or its downstream effectors, are also responsible for effective chloroplast targeting. 

Other GO terms enriched in the *nacβ1nacβ2* proteome were connected to stress response. As was previously reported in plants, NACβ was observed to be important for stress tolerance, usually leading to lower tolerance upon silencing and enhanced tolerance if overexpressed [28,29]. Furthermore, the germination rate of the *nacβ1nacβ2* seeds was slowed down under the lower concentrations of NaCl or mannitol compared to the Col-0 wt (Figure S4). As observed in the flower bud transcriptome, there was also upregulation of chaperones in the *nacβ1nacβ2* proteome. Six of the chaperones were related to de novo protein folding including two chaperonins connected with chloroplast import—CPN60B3 (AT5G56500), and CPN10-1 (AT3G60210)—and three HSP70 family chaperones HSP70-2 (AT5G02490), HSP70-4, and HSP70-8. HSP70-8 and HSP70-4 were also upregulated as transcripts. In mammals, HSP70L1 was shown to be NAC partner in protein folding [68], whereas in yeast, the mutation of HSP70-type chaperones ssb1 and ssb2 was complemented by NAC [5]. Also, HSP70/40 complexes were important for cytoplasmatic de novo protein folding [69]. HSP40 family dnaJ2 (AT5G22060) was also upregulated in the mutant. However, heat stress proteins were downregulated—ZEP (At5g67030) important for ABA synthesis, xanthophyll cycle and photooxidative stress, and Y3IP1 (AT5G44650) responsible for photosystem I assembly and photooxidative stress response. Other stress-related proteins were connected to water deficit, response to toxic substances and oxidative stress, majority of which were downregulated in the mutant. For instance, 6 LEA proteins were among the downregulated in both analyses. 

To summarize transcriptomics and proteomics data together, there were shared 15 upregulated genes/proteins and 98 downregulated ones (Table S5). Despite of the low overlap in the GO terms by themselves, there were observed several similar trends between the transcriptome and the proteome, for instance upregulation of chaperones or down-regulation of stress-related proteins. The absence of DE proteins connected with pollen development (unlike in the transcriptome analyses where there were detected several of them) could be explained by the mRNA storage during pollen maturation and simply absence of yet not-translated proteins, since only 13 proteins encoded by the DEGs connected to pollen development were discovered in the proteome analysis. Seven of these proteins passed the statistical thresholds and were also downregulated in the mutant. The analysis also proposes the role of NACs in regulation of photosynthetic apparatus.

## 4. Materials and Methods 

### 4.1. Plant Cultivation

The seeds of Columbia-0 (wild type), and T-DNA insertion lines SALK_043673 (insert in At1g73230, *NACβ1*), and GK–368H02 (insert in At1g17880, *NACβ2*) were purchased from the Nottingham Arabidopsis Stock Centre (NASC; http://arabidopsis.info, Nottingham, United Kingdom). The double homozygous mutants *(nacβ1nacβ2)* were acquired by a conventional cross of these two T-DNA insertion lines to acquire double heterozygous plants, and then they were self-pollinated to get the double homozygous mutant. The seeds were first sterilized briefly in ethanol, and then 5 min in 0.45% (w/v) sodium hypochlorite with Tween 20. The solution was exchanged for distilled water, and the seeds were washed five times in distilled water to remove residual sodium hypochlorite. Finally, the seeds were placed into 70% ethanol, from which were put onto sterilized filtration paper and were let dry. They were sown either onto plates with MS medium (1% w/v sucrose, 1% w/v agar, 2.3 g MS basal salts from Sigma-Aldrich, M5524 to 1 L media [70]) or directly onto Jiffy tablets. The plant pots/plates were stratified for 3 days in the cold room (4 °C) and then transferred to the cultivation room under long day (16h light/8h dark) conditions at 21 °C. 14 days after sowing, the plants (both from Jiffy tablets, and MS plates) were prickled to the new pots with Jiffy tablets. 

### 4.2. Insertion Lines and Genotyping

The plants were genotyped by PCR. First, the genomic DNA was extracted from leaves of individual plants by a modified CTAB (cetyltrimethylammonium bromide) protocol [71]. This extracted genomic DNA served as a template for two PCR reactions, the first one revealing the wild type allele by the forward and reverse gene-specific primers (Table S1), and the second one detecting insert by a combination of either forward or reverse gene-specific and insert-specific primer (Table S1). The used insert-specific primers were LBb1.3 in the SALK line (SALK_043673), and o8409 in the GK line (GK–368H02). The PCR reactions contained 0.4 μM each primer (Sigma-Aldrich, Haverhill, United Kingdom), 0.2 mM each dNTP (Promega, Madison, USA), 1 μL DNA template (the extracted genomic DNA from leaves), 1U *Taq* DNA polymerase (Merci, Brno, Czech Republic) and 1× reaction buffer according to manufacturer’s instructions. The PCR cycle was carried out as follows: 1) 94 °C, 2 min; 40 cycles: 2) 94 °C, 30 s, 3) 55 °C, 30 s, 4) 72 °C, 1.5 min; 5) 72 °C, 5 min. Post PCR, the reaction was blended with 6× Loading Dye (Thermo Fisher Scientific, Waltham, USA) and its aliquot was electrophoresed in 1.2% (w/v) agarose gel with ethidium bromide in 1× TAE buffer (40 mM Tris, 20 mM acetic acid, 1 mM EDTA [72]). GeneRuler 100 bp Plus DNA Ladder (Thermo Fisher Scientific, Waltham, USA) was used as a molecular marker. The gels were imaged by Gel Documentation System G-Box (Syngene, Cambridge, United Kingdom).

### 4.3. Phenotype Characterization

Several phenotypic traits of the *nacβ1nacβ2* plants were compared to Col-0 wild type plants. These observations were performed in three independent sample batches considered as biological replicates. (i) The chlorophyll content in the leaves was determined according to Witham et al. (1971) [73] with slight modifications. Briefly, 0.1 g fresh weight of leaves were grinded by a pestle in a mortar with 5 mL 80% (v/v) acetone. The extract was filtered, and the sample was topped up to 10 mL with 80% (v/v) acetone. The optical density of the extracted samples was measured by a spectrophotometer (BioMate 3, Thermo Fisher Scientific, Waltham, USA) blanked with 80% acetone in 10 mm thin cuvettes at 645 nm, 652 nm, and 663 nm. Finally, chlorophyll content was calculated according to the formulas given in Witham et al. (1971) [73]. (ii) Flower buds, which were maximum one day before anthesis, were dissected and observed under the dissection microscope and the numbers of flower organs (sepals, petals, and anthers) were calculated and categorized. (iii) The length of green immature siliques on the main stem was measured by a paper ruler. (iv) The number of seeds inside the siliques was determined as follows. The green immature siliques were cut from the plant, stuck to the glass slide by a double-sided tape, and opened by an injection needle. The number of developed green seeds, pale normal-sized seeds, and aborted seeds was calculated under the dissection microscope (Zeiss Stemi 508, Oberkochen, Germany). The graphs were constructed in MS Excel (Microsoft, Redmond, USA).

### 4.4. Transmission Analysis

The heterozygous plants in one *NACβ* gene on the mutant background of the other one (*nacβ1/+ nacβ2*, and *nacβ1nacβ2/+*; + symbolizes heterozygous gene) were let self-pollinate. Their seeds were cultivated as above and genotyped by PCR under the conditions as above. From the offspring genotype, the transmission coefficients were calculated. Next, several crosses were performed. Firstly, the heterozygous plants in one *NACβ* gene on the mutant background of the other one (*nacβ1/+nacβ2*, and *nacβ1nacβ2/+*, respectively) were crossed with Col-0 wild type plants in both cases (carrying two wild type alleles of each *NACβ* gene). Secondly, the heterozygous plants in one *NACβ* gene on the mutant background of the other one (*nacβ1/+nacβ2*, *nacβ1nacβ2/+*, respectively) were crossed with plants representing the same gene as a homozygous mutant (*nacβ2*, and *nacβ1*, respectively). All the mentioned crosses were carried reciprocally, plants of each genotype serving once as the donor of the pistils and the second time donating pollen grains. The crosses were performed as follows; the flower buds before anther dehiscence of the pistil donors were emasculated and the pistils were let to mature for 1–2 days. After their papillary cells were developed, they were pollinated with the desired pollen. The acquired seeds were sown and cultivated as above, and the plants were conventionally genotyped by PCR using the primers as above. From the offspring genotype, the transmission coefficients were calculated, and χ^2^-test was performed.

### 4.5. Pollen Tube Cultivation in vitro

Pollen tube cultivation in vitro was carried out according to Boavida and McCormick (2007) [34]. Briefly, the cultivation medium (0.01% (w/v) H_3_BO_3_, 1 mM CaCl_2_, 1 mM KCl, 5% (w/v) sucrose, pH 7.5, 1% (w/v) low-melting agarose) was dropped onto the glass slide and let solidify. The tested pollen grains were shed from the open flowers onto the medium and the slides were incubated inside the small boxes in the cultivation room. After 8-hours growth, the pollen tubes were observed under the inverted epifluorescent microscope (Nikon TE2000E, Tokyo, Japan), and the images analyzed by NIS elements (Nikon, Tokyo, Japan).

### 4.6. Pollen Tube Cultivation in vivo and Aniline Blue Staining

The young flower buds were emasculated before anther dehiscence. The pistils were let to develop papillary cells for 1–2 days, and then were pollinated. The pollen tubes were let grow overnight. The pistils were stained by aniline blue according to Mori et al. (2006) [74]. Briefly, the harvested pistils were fixed for 2 hours in ethanol with acetic acid (3:1), and subsequently hydrated in ethanol series (70%, 50%, and 30% ethanol). After an overnight incubation in 8 M NaOH to allow tissue softening, the callose in the cell walls of the pollen tubes was stained by 0.1% (w/v) aniline blue in 108 mM K_3_PO_4_ (pH 11) and 2% (v/v) glycerol. Prior to the observation under epifluorescent microscope (Nikon TE2000E, Tokyo, Japan), the samples were transferred to distilled water.

### 4.7. Blue Dot Assay

The blue dot assay was performed as described previously [37]. Briefly, the young flower buds before anther dehiscence were emasculated, and the pistils were let mature for 1–2 days. The prepared pistils were pollinated with wild type pollen grains carrying pLAT52–GUS construct. The pistils were collected 24 hours after pollination, dissected under dissection microscope, and transferred to the GUS staining solution (50 mM sodium phosphate buffer, pH 7.0, 0.2% Triton X-100, 10 mM potassium ferrocyanide, 10 mM potassium ferricyanide, and 1 mM X-Gluc [5-bromo-4-chloro-3-indolyl-D-glucoronic acid]). After overnight staining at 37 °C, the samples were observed under the epifluorescent microscope (Nikon TE2000E, Tokyo, Japan) in the bright field.

### 4.8. Cloning

The cloned DNA fragments were amplified from genomic DNA (extracted by CTAB as above for plant genotyping). At first, the DNA fragments were synthesized by a 2-step PCR with Phusion High-Fidelity DNA Polymerase (Thermo Fisher Scientific, Waltham, USA) according to the manufacturer’s instructions. The DNA fragments for cloning were synthesized by PCR primed by the primers (Sigma-Aldrich, Haverhill, United Kingdom) in Table S1. The second PCR reaction was in all cases except for the DD33 promoter primed by the AttB1 and AttB2 primer pair. The DD33 promoter was in the second PCR reaction amplified by the primer pair AttB4 and AttB1R. The amplified sequences except for the DD33 promoter bordered by attB1/B2-overhangs were recombined by Gateway™ BP Clonase™ II Enzyme mix (Thermo Fisher Scientific, Waltham, USA) to pDONR221 (Thermo Fisher Scientific, Waltham, USA), thus creating entry clones. The DD33 promoter with attB4/1R overhangs was recombined by BP reaction under the same conditions into the vector pDONRP4-P1r (Thermo Fisher Scientific, Waltham, USA). The vectors were transformed to self-prepared chemically competent *E. coli* of the TOP10 strain. After plasmid isolation by GeneJET Plasmid Miniprep Kit (Thermo Fisher Scientific, Waltham, USA), and verification by Sanger sequencing, the fragments were recombined by Gateway™ LR Clonase™ II Enzyme mix (Thermo Fisher Scientific, Waltham, USA) to the following destination vectors. The overexpression constructs tagged with GFP were formed from vectors pFAST-R05 (C-terminal GFP fusion), and pFAST-R06 (N-terminal GFP fusion) [75]. The constructs for complementation analysis and protein localization were derived from pB7FWG,0 (https://gateway.psb.ugent.be, Ghent, Belgium [76]) whereas the constructs for promoter activity were created from pKGWFS7,0 (https://gateway.psb.ugent.be, Ghent, Belgium [76]). The overexpression experiments driven by LAT52 [39] and DD33 [40] promoters were carried out by destination vector pB7m34GW (Thermo Fisher Scientific, Waltham, USA) putting three fragments together. For LAT52 overexpression, pDONRP4-P1r with LAT52 promoter donated by David Twell’s laboratory was recombined with pDONR221 carrying the coding genomic sequence of the *NACβ* genes, and with pDONRP2r-P3 with GFP sequence donated by David Twell’s laboratory. For DD33 overexpression, pDONRP4-P1r with DD33 promoter prepared as above was recombined with pDONR221 carrying the coding genomic sequence of the *NACβ* genes, and with pDONRP2r-P3 with GFP sequence donated by David Twell’s laboratory. The acquired destination vectors were again transformed to self-prepared chemically competent *E. coli* of the TOP10 strain. The vectors were isolated from the selected *E. coli* suspensions by GeneJET Plasmid Miniprep Kit (Thermo Fisher Scientific, Waltham, USA) and subsequently verified by Sanger sequencing.

### 4.9. Arabidopsis Thaliana Transformation and Microscopy

The *Agrobacterium tumefaciens* strain GV3101 (homemade complementary cells) was transformed by electroporation with all the isolated constructs, and subsequently *Arabidopsis thaliana* wild type (protein localization, overexpression, promoter analysis) or *nacβ1/+nacβ2*, and *nacβ1nacβ2/+* (complementation analysis) flowers were dipped into *A. tumefaciens* cell suspension to acquire stable transformants [77], which were selected either on MS medium [70] with proper selection (BASTA 10 μg·μL^−1^; vector pB7FWG, 0 for protein localization and complementation analysis, and vector pB7m34GW for proDD33- and proLAT52-driven overexpression or kanamycin 50 μg·μL^−1^; vector pKGWFS7,0 for promoter analysis) or by screening for the red seeds under fluorescent microscope (vectors pFAST-R05, and pFAST-R06 for pro35S-driven overexpression). The presence of the inserts in the transformed plants was verified by PCR with GFP forward and reverse primer pair. Then, the fluorescent signal was checked under the epifluorescent inverted microscope (Nikon TE2000E, Tokyo, Japan). The best transformants were selected and observed in more detail under confocal fluorescent microscope (Zeiss LSM 880 with Airyscan detector, Oberkochen, Germany).

### 4.10. Promoter Analysis

The promoter analysis was performed by constructs tagged with both GFP and GUS. The transformants were selected according to the presence of the insert detected by PCR as above and according to the presence of GFP signal under the epifluorescent microscope. The selected plants were screened for GUS signal [78]. Briefly, various tissue types (seedlings, leaves, cauline leaves, inflorescences, flower buds, flowers, pollen grains, siliques) were incubated usually for 2 h (in case of low signal, the duration was prolonged to 24 h) in GUS staining buffer (100 mM sodium phosphate buffer pH 7.0, 10 mM EDTA, 0.1% (v/v) Triton X-100, 0.5 mM potassium ferrocyanide, 0.5 mM potassium ferricyanide, and 1 mM X-Gluc), and then bleached with ethanol series (90%, 70%, 50%). The tissues from several representative plants were observed and photographed under the dissection microscope (Zeiss Stemi 508, Oberkochen, Germany).

### 4.11. RNA Extraction, cDNA Library Preparation and Sequencing

The total RNA was extracted from harvested *A. thaliana* young flower buds (up to stage 12 [43]) of both single *NACβ* homozygous mutants (*nacβ1*, and *nacβ2*) together with double *NACβ* homozygous mutant (*nacβ1nacβ2*) and control wild type Col-0 plants (genotype *NACβ1NACβ2*) using the RNeasy Plant Mini Kit (Quigen, Venlo, Netherlands) according to the manufacturer’s instructions. Collected RNA was treated with DNA-*free*^TM^ DNA Removal Kit (Thermo Fisher Scientific, Waltham, USA) and the integrity, purity and concentration of samples was measured on bioanalyzer (IMG-CORE-AGILENT 2100, Santa Clara, USA). Three biological replicates of each genotype were used for sequencing. 3 μg of high-quality RNA was used for library preparation. The libraries were prepared for paired-end sequencing by TruSeq Stranded mRNA LT Sample Prep Kit and TruSeq Stranded Total RNA LT Sample Prep Kit (Plant) following the TruSeq Stranded mRNA Sample Preparation Guide, Part # 15031047 Rev. E and TruSeq Stranded Total RNA Sample Prep Guide, Part # 15031048 Rev. E and sequenced on Illumina platform (Illumina, California, USA) by Macrogen Inc. (Seoul, Republic of Korea). The data were deposited into BioProject ID PRJNA589533 (https://www.ncbi.nlm.nih.gov/bioproject/589533).

### 4.12. Mapping and Assembly Of Reads

Raw reads were further processed as follows. The quality of pair-end raw reads was revised by FastQC ver. 0.11.8 [79] (http://www.bioinformatics.babraham.ac.uk/projects/fastqc), and Cutadapt ver. 1.9.1 [80]. The quality reads (phred score > 20) were also trimmed of technical sequences using the same Cutadapt software. Paired-reads with at least one read shorter than 20 bp were also excluded. Consequently, the reads were mapped to reference genome of *A. thaliana* (ver. TAIR10) downloaded from the Araport database (https://www.araport.org/downloads/TAIR10_genome_release) by STAR software ver. 206.1a [81] and then, the reads were counted by the featureCounts program from the Subread package ver. 1.6.3 [82]. Differential expression analyses were performed with the DESeq2 software ver. 3.8 [83] with adjusted p-value < 0,05 and FoldChange ≥ ±2 used as thresholds for establishing significantly differentially expressed genes (DEGs).

### 4.13. Annotation and Enrichment Analyses

To bring deeper insight into the functional significance of DEGs, GO enrichment analyses were performed using Panther database (http://pantherdb.org, annotation version: GO Ontology database Released 2019-07-03) using Fisher´s exact test with false discovery rate <0,05 as statistical significance threshold. ThaleMine, tool of Araport website, was used for functional annotation of DEGs list (https://apps.araport.org/thalemine/begin.do, ver. 1.10.4). To identify molecular pathways, list of DEGs was further analyzed using the KEGG database (https://www.genome.jp/kegg, Release 89.0). 

### 4.14. Confirmation of DEGs Expression by qRT-PCR

Genes encoding NACβ subunits (At1g17880, At1g73230), NACα subunits (At3g12390, At3g49470, At5g13850, At4g10480, At1g33040) and five other genes (At5g64120, At2g20142, At5g52390, At2g43510, At3g12580) were chosen for qRT-PCR verification. EARLY FLOWERING 4 (eLF4; At2G40080) and tubulin beta chain 3 (tub3; At5g62700) were added as references. Residual RNA collected for sequencing of each plant genotype (*nacβ2*, *nacβ1*, *nacβ1nacβ2, NACβ1NACβ2*) was used for the qRT-PCR in three biological replicas. The reactions were prepared with GoTaq® qPCR Master Mix (Promega, Madison, WI, USA) and analyzed on LightCycler 480 Instrument (Roche, Basel, Switzerland) according to the manufacturer’s instructions using the primer sets from Table S2. cT values of each sample were recorded by the LightCycler 480 Software version 1.5 (Roche, Basel, Switzerland). Quality control of the obtained data was performed by the same software. Fragments per kilobase million (FPKM) values of transcript from RNA sequencing (RNA-seq) data were calculated with RSEM software [84]. Linear regression of cT values and log based FPKM values was projected in MS Excel (Microsoft, Redmond, USA).

### 4.15. Protein Extraction and Proteome Analysis

The total proteins were extracted from the harvested *A. thaliana* young flower buds (up to stage 12 [43]) of double *NACβ* homozygous mutant (*nacβ1nacβ2*) and control wild type Col-0 plants (genotype *NACβ1NACβ2*) by TRI-reagent (Sigma-Aldrich, St. Louis, USA) according to the manufacturer’s instructions with slight modifications [85]. Briefly, 45 mg flower buds were snap-frozen in liquid nitrogen and then homogenized by a pestle in a mortar. To the grinded plant material, TRI reagent was added stepwise per 200 µL to achieve a final volume of 1 mL to extract the proteins. Then, 200 µL chloroform was added, and the sample was let on ice for 15 min. After centrifugation (20,000×g, 20 min, 4 °C), the lower phase was mixed with 300 µL ethanol. After another round of centrifugation (2,000×g, 5 min, 4 °C), the supernatant was mixed with 1.5 volumes isopropanol and the proteins were let precipitate at room temperature for 1 h. The proteins were pelleted (20,000×g, 20 min, 15 °C), washed 3 times with 1 mL cold ethanol and centrifuged again (20,000×g, 20 min, 4 °C). The final protein pellet was vacuum dried and stored at −20 °C until further use.

Dried pellets were solubilized by SDT buffer (4% SDS, 0.1 M DTT, 0.1 M Tris-HCl, pH 7.6) and protein concentration measured by native tryptophan fluorescence. Fifty micrograms of proteins were used for filter-aided sample preparation (FASP) method [86] with some modifications. The samples were mixed with 8M UA buffer (8 M urea in 100 mM Tris-HCl, pH 8.5), loaded onto the Microcon device with MWCO 30 kDa (Merck Millipore, Burlington, Massachusetts, USA) and centrifuged (7,000×g, 30 min, 20 °C). The retained proteins were washed (all centrifugation steps after sample loading were performed at 14,000× g) with 200 μL UA buffer. The washed protein concentrates kept in the Microcon device were mixed with 100 μL of UA buffer containing 50 mM iodoacetamide and incubated in the dark for 20 min. After the next centrifugation step, the samples were washed three times with 100 μL UA buffer and three times with 100 μL of 50 mM NaHCO_3_. Trypsin (sequencing grade, Promega, Madison, USA) was added onto the filter and the mixture was incubated for 18 h at 37 °C (enzyme:protein ratio 1:50). The tryptic peptides were eluted by centrifugation followed by two additional elutions with 50 μL of 50 mM NaHCO_3_. Peptides were extracted into LC-MS vials by 2.5% formic acid (FA) in 50% acetonitrile (ACN) and 100% ACN with addition of polyethylene glycol (20,000; final concentration 0.001%; [87]) and concentrated in a SpeedVac concentrator (Thermo Fisher Scientific, Waltham, USA) prior to LC-MS/MS analyses.

LC-MS/MS analyses of all peptide mixtures were performed by RSLCnano system (SRD-3400, NCS-3500RS CAP, WPS-3000 TPL RS) connected to Orbitrap Q Exactive HF-X spectrometer (Thermo Fisher Scientific, Waltham, USA). Prior to LC separation, tryptic digests were online concentrated and desalted by trapping column (Acclaim™ PepMap™ 100 C18, dimensions 300 μm × 5 mm, 5 μm particles; part number 160454). After washing of trapping column with 0.1% FA, the peptides were eluted in backflush mode (flow 300 nL.min^−1^) from the trapping column onto an analytical column (Acclaim Pepmap100 C18, 3 µm particles, 75 μm × 500 mm; Thermo Fisher Scientific, Waltham, USA) by 100 min gradient program (2%–35% of mobile phase B; mobile phase A: 0.1% FA in water; mobile phase B: 0.1% FA in 80% ACN). Equilibration of the trapping column and the column was done prior to sample injection to sample loop. The analytical column outlet was directly connected to the Digital PicoView 550 (New Objective) ion source with sheath gas option and SilicaTip emitter (New Objective; FS360-20-15-N-20-C12) utilization. ABIRD (Active Background Ion Reduction Device, ESI Source Solutions) was installed.

MS data were acquired in a data-dependent strategy selecting up to top 20 precursors based on precursor abundance in the survey scan (350–2000 m/z). The resolution of the survey scan was 120 000 (200 m/z) with a target value of 3×10^6^ ions and maximum injection time of 50 ms. HCD MS/MS (28% relative fragmentation energy) spectra were acquired with a target value of 10,000 and resolution of 15,000 (200 m/z). The maximum injection time for MS/MS was 50 ms. Dynamic exclusion was enabled for 40 s after one MS/MS spectra acquisition. The isolation window for MS/MS fragmentation was set to 1.2 m/z.

The analysis of the mass spectrometric RAW data files was carried out by the MaxQuant software (version 1.6.2.10) using default settings unless otherwise noted. MS/MS ion searches were performed against modified cRAP database (based on http://www.thegpm.org/crap) containing protein contaminants like keratin, trypsin etc., and UniProtKB protein database for *Arabidopsis thaliana* (ftp://ftp.uniprot.org/pub/databases/uniprot/current_release/knowledgebase/reference_proteomes/Eukaryota/UP000006548_3702.fasta.gz; downloaded 8. 7. 2019, version 2019/07, number of protein sequences 27,476). Oxidation of methionine and proline, deamidation (N, Q) and acetylation (protein N-terminus) as optional modification, carbamidomethylation (C) as fixed modification and trypsin/P enzyme with 2 allowed miss cleavages were set. Peptides and proteins with FDR threshold < 0.01 and proteins having at least one unique or razor peptide were considered only. Match between runs was set among all analyzed samples. Protein abundance was assessed according to protein intensities calculated by MaxQuant.

Protein intensities reported in proteinGroups.txt were further evaluated using the software container environment (https://github.com/OmicsWorkflows/KNIME_docker_vnc; version 3.7.2a). Processing workflow is available upon request and it covered decoy hits and removal of contaminant protein groups, protein group intensities log_2_ transformation, normalization, imputation of missing values (imp4p R package) and statistical analysis (LIMMA; p values adjustment using Benjamini and Hochberg approach). The final list of differently expressed proteins was acquired according to the following criteria: the fold change > 2, adjusted p value < 0.05; protein groups had to have at least 2 peptides and non-zero protein group intensity in at least 2 replicates of at least one sample.

The mass spectrometry proteomics data have been deposited to the ProteomeXchange Consortium via the PRIDE partner repository with the dataset identifier PXD016315.

### 4.16. Plant Cultivation Under The Salt and Osmotic Stress

The seeds of Columbia-0 (wild type), and double *NACβ* homozygous mutant (*nacβ1nacβ2*) were sterilized as above and sown onto the plates with MS medium (1% w/v sucrose, 1% w/v agar, 2.3 g MS basal salts from Sigma-Aldrich, M5524 to 1 L media [70] supplemented either with 50 mM, 100 mM, 125 mM, and 150 mM NaCl, respectively or 250 mM, 300 mM, and 350 mM mannitol, respectively. The plates were stratified in the cold room for 1 day and then transferred to the cultivation room. The proportion of grown seedlings was calculated 4, 6, 8, 11, and 13 days after the transfer to the cultivation room. 

## 5. Conclusions

Collectively, this paper presented the functional analysis of *nacβ1nacβ2* mutants and proposed the role of the β-subunit of the nascent polypeptide-associated complex during flower and silique development of *Arabidopsis thaliana*. Moreover, the flower bud transcriptomic and proteomic data suggested that NACβ subunits are likely involved in stress responses and male gametophyte development. The role of NAC complex during stress is consistent with the data acquired previously on different plant species and our experimental data. 

## Figures and Tables

**Table 1:** The transmission efficiency of the *nacβ1* and *nacβ2* alleles in self-crosses of the heterozygous plants on the mutant background of the other *NACβ* allele (*nacβ1/+;nacβ2* or *nacβ1;nacβ2/+*, respectively). χ^2^ column shows the p-value of the χ^2^ test.

Parent	Selfing Offspring	Expected Mendelian Ratio	Observed Ratio	Change in Transmission Efficiency	χ^2^
nacβ1/+;nacβ2 *NACβ1* transmission (*N* = 119)	*NACβ1nacβ2*	29.75	33	+11%	7.5 × 10^−2^
	*nacβ1/+;nacβ2*	59.50	67	+13%	
	*nacβ1nacβ2*	29.75	19	−46%	
nacβ1;nacβ2/+ *NACβ2* transmission (*N* = 115)	*nacβ1NACβ2*	28.75	37	+29%	8.8 × 10^−3^
	*nacβ1;nacβ2/+*	57.50	63	+10%	
	*nacβ1nacβ2*	28.75	15	−48%	

**Table 2:** The transmission efficiency of the *nacβ1* and *nacβ2* alleles in out-crosses of the heterozygous plants (*nacβ1/+;nacβ2* or *nacβ1;nacβ2/+*, respectively) with Col-0 (wt) via both female and male gametophyte. χ^2^ column shows the p-value of the χ^2^ test. 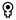 means female, whereas 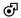 means male.

Cross	Mendelian Rate of Mutant Allele	Observed Rate of Mutant Allele	Mutant Allele Transmission Efficiency—Wild Type Background		χ^2^
*nacβ1/+;nacβ2* 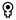 × Col-0 (wt) 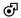 (*N* = 88)	44	53	*nacβ1* 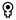	+20%	5.5 × 10^−2^
*nacβ1;nacβ2/+* 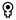 × Col-0 (wt) 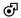 (*N* = 118)	59	50	*nacβ2* 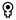	−15%	9.8 × 10^−2^
Col-0 (wt) 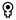 × *nacβ1/+;nacβ2* 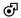 (*N* = 128)	64	97	*nacβ1* 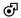	+52%	5.4 × 10^−9^
Col-0 (wt) 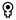 × *nacβ1;nacβ2/+* 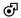 (*N* = 133)	66.5	52	*nacβ2* 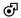	−22%	1.2 × 10^−2^

**Table 3:** The transmission efficiency of the *nacβ1* and *nacβ2* alleles in out-crosses of the heterozygous plants (*nacβ1/+;nacβ2* or *nacβ1;nacβ2/+*, respectively) in the mutant background of the other *NACβ* gene via both female and male gametophyte. χ^2^ column shows the p-value of the χ^2^ test. 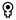 means female, whereas 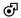 means male.

Cross	Mendelian Rate of Mutant Allele	Observed Rate of Mutant Allele	Mutant allele Transmission Efficiency—Mutant Background		χ^2^
*nacβ1/+;nacβ2* 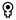 × *NACβ1nacβ2* 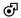 (*N* = 114)	57	54	*nacβ1* 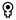	−5%	5.7 × 10^−1^
*nacβ1;nacβ2/+* 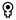 × *nacβ1NACβ2* 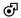 (*N* = 143)	71.5	59	*nacβ2* 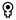	−17%	3.7 × 10^−2^
*NACβ1nacβ2* 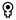 × *nacβ1/+;nacβ2* 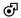 (*N* = 142)	71	70	*nacβ1* 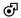	−1%	8.7 × 10^−1^
*nacβ1NACβ2* 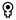 × *nacβ1;nacβ2/+* 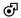 (*N* = 140)	70	58	*nacβ2* 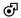	−17%	4.3 × 10^−2^

## Supplementary Materials

Supplementary materials can be found at https://www.mdpi.com/1422-0067/21/6/2065/s1.

## Abbreviations

*ag*	*agamous*
*ap3*	*apetala 3*
BASTA	(RS)-2-Amino-4-(hydroxy(methyl)phosphonoyl)butanoic acid
bp	base pair
Col-0	Columbia-0
CaMV	cauliflower mosaic virus
*clv1*	*clavata 1*
*clv3*	*clavata 3*
CTAB	cetyltrimethylammonium bromide
DE	differential expression
DEG	differentially expressed gene
DEP	differentially expressed protein
dNTP	deoxyribonucleotide triphosphate
EDTA	ethylenediaminetetraacetic acid
GFP	green fluorescent protein
FPKM	fragments per kilobase million
GO	gene ontology
GUS	glucuronidase
HSP	heat shock protein
MS	Murashige and Skoog growth medium
NAC	nascent polypeptide-associated complex
*nacβ1nacβ2*	NACβ double homozygous mutant
PCR	polymerase chain reaction
*pi*	*pistillata*
qRT-PCR	quantitative reverse transcriptase polymerase chain reaction
RNA-seq	RNA sequencing
*sep*	*sepallata*
TAE	electrophoresis buffer composed of Tris base, acetic acid and EDTA
Tris	2-Amino-2-(hydroxymethyl)propane-1,3-diol
*wig*	*wiggum*
wt	wild type
