# Supplementary material for: The Beta Subunit of Nascent Polypeptide Associated Complex Plays A Role in Flowers and Siliques Development of Arabidopsis thaliana
Source: Int J Mol Sci. 2020 Mar 17;21(6):2065. doi: 10.3390/ijms21062065 (PMC7139743; doi:10.3390/ijms21062065)
Supplement: Supplementary file 1 [file ijms-21-02065-s001.zip › table_S5.docx]

|  | | transcriptome | | total |
| --- | --- | --- | --- | --- |
|  |  | up-regulated | down-reguated |  |
| proteome | up-regulated | 15 | 1 | 170 |
|  | down-regulated | 0 | 98 | 290 |
| total | | 363 | 1602 |  |
