# Supplementary material for: The Beta Subunit of Nascent Polypeptide Associated Complex Plays A Role in Flowers and Siliques Development of Arabidopsis thaliana
Source: Int J Mol Sci. 2020 Mar 17;21(6):2065. doi: 10.3390/ijms21062065 (PMC7139743; doi:10.3390/ijms21062065)

**Table of contents**

**Supplementary table legends**

**Table S1 – Table of primers used for genotyping of the insertion lines and for cloning of NACβ genomic fragments.**

**Table S2 – Table of primers used for qRT-PCR amplification.**

**Table S3 – Table of all differentially expressed genes in *nacβ1nacβ2.*** The table consists of 13 columns as follows: Gene ID, fold change, fold change in log_2_ scale, adjusted p-value, symbol of gene and brief description obtained from Araport database and GO terms for gene groups mentioned in the article. Genes are sorted according to their log_2_Fold Change.

**Table S4 – Table of all differentially expressed proteins in *nacβ1nacβ2.*** The table consists of 20 columns as follows: Protein group accession number, Gene ID, symbol, Brief Description obtained from v1.10.4, Araport11 Release 06/2016 database, logFC, FC, adjusted p-value, p-value and GO terms for protein groups mentioned in the article. Proteins are sorted according log_2_Fold Change.

**Table S5 – Table of comparison of DE genes and proteins in *nacβ1nacβ2* proteome and transcriptome.** The table shows numbers of differentially expressed proteins/genes present in both analyses as well as total number of discovered upregulated/downregulated proteins and genes.

**Supplementary figure legends**

**Figure S1 – Development of *nacβ1nacβ2* and Col-0 wt, and characterization of *nacβ* single mutant insertion lines.** A–C Comparison of the *nacβ1nacβ2* and Col-0 wt development under the same conditions. The first six rows of plants are represented by *nacβ1nacβ2* whereas the last three rows of plants are represented by Col-0 wt. A – 35 days after sowing. B – 41 days after sowing. C – 52 days after sowing. D – The scheme showing the position of the insert (indicated by grey triangle) in the T-DNA insertion line SALK_043673 (in NACβ1). Arrows indicate the positions of primers used for genotyping. E – The scheme showing the position of the insert (indicated by grey triangle) in the T-DNA insertion line GK368-H02 (in NACβ2). Arrows indicate the positions of primers used for genotyping. F – Linear regressions of log_2_FPKM values obtained from RNA-seq data and cT values from RT-qPCR for NACβ1 (At1g73230), and NACβ2 (At1g17880) with correlation coefficient R^2^=0.9645 (NACβ1) or R^2^=0.6209 (NACβ2), respectively.

**Figure S2 – Flower and silique phenotype of the *nacβ1nacβ2* plants.** A – Column chart showing the distribution of various phenotype categories of *nacβ1nacβ2* flowers. Wild type flowers are highlighted in black. A total of 202 flowers was observed. C stands for petals, K for sepals, and A for anthers. The number of gynoecia was the same as in wt flowers. B – Box-and-whisker plot showing the median and quartiles revealing the silique length of the *nacβ1nacβ2*, and Col-0 wt. Both datasets were statistically compared by Student’s t-test, p-value of which is given. C – Box-and-whisker plot showing the median and quartiles revealing the number of seeds per silique. Both datasets were statistically compared by Student’s t-test, p-value of which is given. D – Column chart showing the proportion of viable green seeds in *nacβ1nacβ2* and Col-0 wt. Both datasets were statistically compared by Student’s t-test, p-value of which is given.

**Figure S3 –** **Validation of RNAseq data with qRT-PCR**. Linear regression of log_2_FPKM values obtained from RNA-seq data and cT values from RT-qPCR for each genotype for twelve DE genes in *nacβ1nacβ2 (*At1g17880, At1g73230, At2g20142, At5g64120, At2g43510, At3g12580, At5g52390, At3g12390, At3g49470, At5g13850, At4g10480, At1g33040) with correlation coefficient R^2^ = 0.7842.

**Figure S4 – Germination of *nacβ1nacβ2* under salt and osmotic stress.** A – Germination rate of Col-0 wt compared to *nacβ1nacβ2* under salt stress caused by 50 mM, 100mM, 125 mM, and 150 mM NaCl. Error bars represent ±5%. The calculations of germinated seeds were performed 4, 6, 8, 11, and 13 days after seed sowing. B – Germination rate of Col-0 wt compared to *nacβ1nacβ2* under osmotic stress caused by 250 mM, 300mM, and 350 mM mannitol. Error bars represent ±5%. The calculations of germinated seeds were performed 4, 6, 8, 11, and 13 days after seed sowing. C – A representative plate of seedlings 13 days after sowing on 125 mM NaCl. D – A representative plate of seedlings 13 days after sowing on 150 mM NaCl. E – A representative plate of seedlings 13 days after sowing under control conditions without added stress agent.

**Table S1 – Table of primers used for genotyping of the insertion lines and for cloning of NACβ genomic fragments.**

**Table S2 – Table of primers used for qRT-PCR amplification.**

**Table S3 – Table of all differentially expressed genes in *nacβ1nacβ2.*** The table consists of 13 columns as follows: Gene ID, fold change, fold change in log_2_ scale, adjusted p-value, symbol of gene and brief description obtained from Araport database and GO terms for gene groups mentioned in the article. Genes are sorted according to their log_2_Fold Change.

**Table S4 – Table of all differentially expressed proteins in *nacβ1nacβ2.*** The table consists of 20 columns as follows: Protein group accession number, Gene ID, symbol, Brief Description obtained from v1.10.4, Araport11 Release 06/2016 database, logFC, FC, adjusted p-value, p-value and GO terms for protein groups mentioned in the article. Proteins are sorted according log_2_Fold Change.

Tables S1–S4 are available as *.xls files.

**Table S5 – Table of comparison of DE genes and proteins in *nacβ1nacβ2* proteome and transcriptome.** The table shows numbers of differentially expressed proteins/genes present in both analyses as well as total number of discovered upregulated/downregulated proteins and genes.

|  | | transcriptome | | total |
| --- | --- | --- | --- | --- |
|  |  | up-regulated | down-reguated |  |
| proteome | up-regulated | 15 | 1 | 170 |
|  | down-regulated | 0 | 98 | 290 |
| total | | 363 | 1602 |  |

**Supplementary figure legends**

**Figure S1 – Development of *nacβ1nacβ2* and Col-0 wt, and characterization of *nacβ* single mutant insertion lines.** A–C Comparison of the *nacβ1nacβ2* and Col-0 wt development under the same conditions. The first six rows of plants are represented by *nacβ1nacβ2* whereas the last three rows of plants are represented by Col-0 wt. A – 35 days after sowing. B – 41 days after sowing. C – 52 days after sowing. D – The scheme showing the position of the insert (indicated by grey triangle) in the T-DNA insertion line SALK_043673 (in *NACβ1*). Arrows indicate the positions of primers used for genotyping. E – The scheme showing the position of the insert (indicated by grey triangle) in the T-DNA insertion line GK368-H02 (in *NACβ2*). Arrows indicate the positions of primers used for genotyping. F – Linear regressions of log_2_FPKM values obtained from RNA-seq data and cT values from RT-qPCR for *NACβ1* (At1g73230), and *NACβ2* (At1g17880) with correlation coefficient R^2^=0.9645 (*NACβ1*) or R^2^=0.6209 (*NACβ2*), respectively.

**
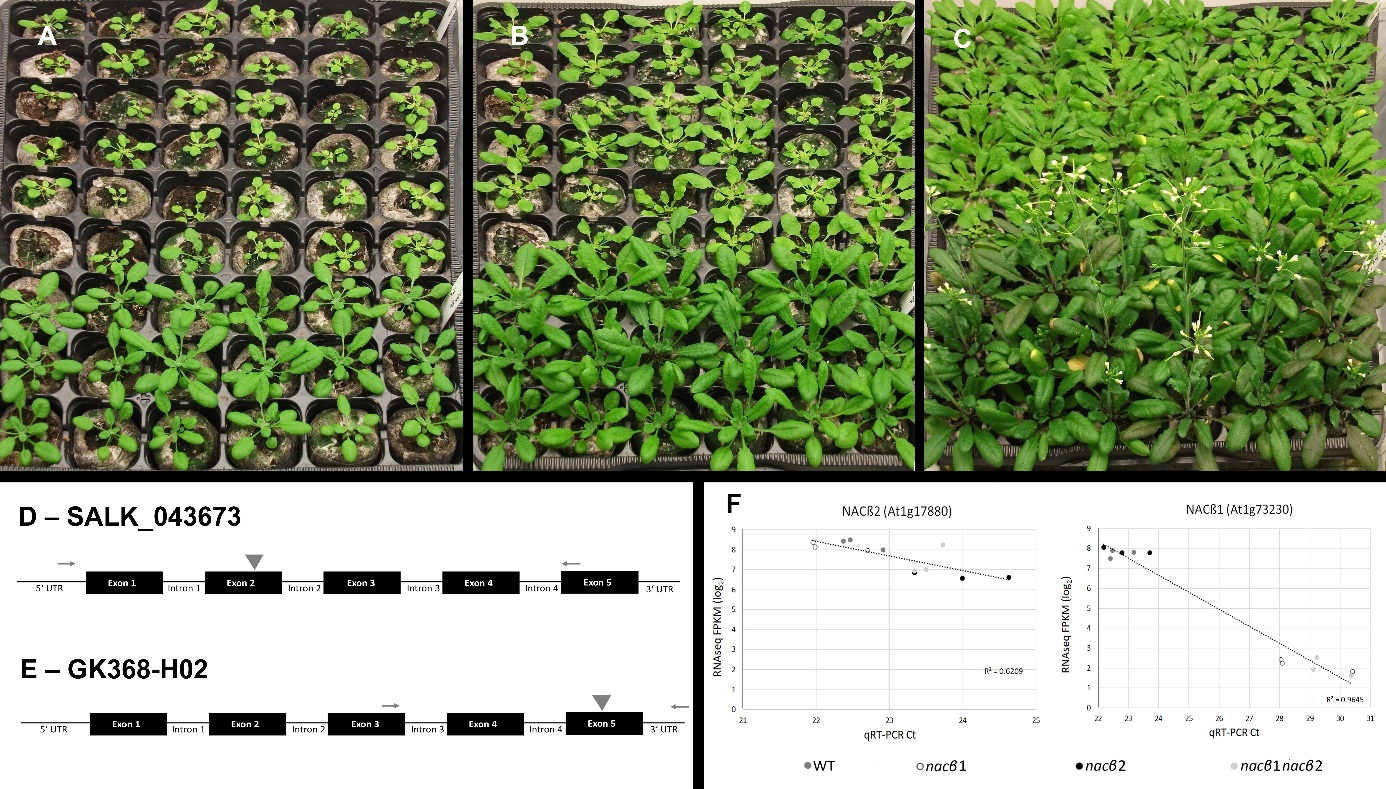
**

**Figure S2 – Flower and silique phenotype of the *nacβ1nacβ2* plants.** A – Column chart showing the distribution of various phenotype categories of *nacβ1nacβ2* flowers. Wild type flowers are highlighted in black. A total of 202 flowers was observed. C stands for petals, K for sepals, and A for anthers. The number of gynoecia was the same as in wt flowers. B – Box-and-whisker plot showing the median and quartiles revealing the silique length of the *nacβ1nacβ2*, and Col-0 wt. Both datasets were statistically compared by Student’s t-test, p-value of which is given. C – Box-and-whisker plot showing the median and quartiles revealing the number of seeds per silique. Both datasets were statistically compared by Student’s t-test, p-value of which is given. D – Column chart showing the proportion of viable green seeds in *nacβ1nacβ2* and Col-0 wt. Both datasets were statistically compared by Student’s t-test, p-value of which is given.

**
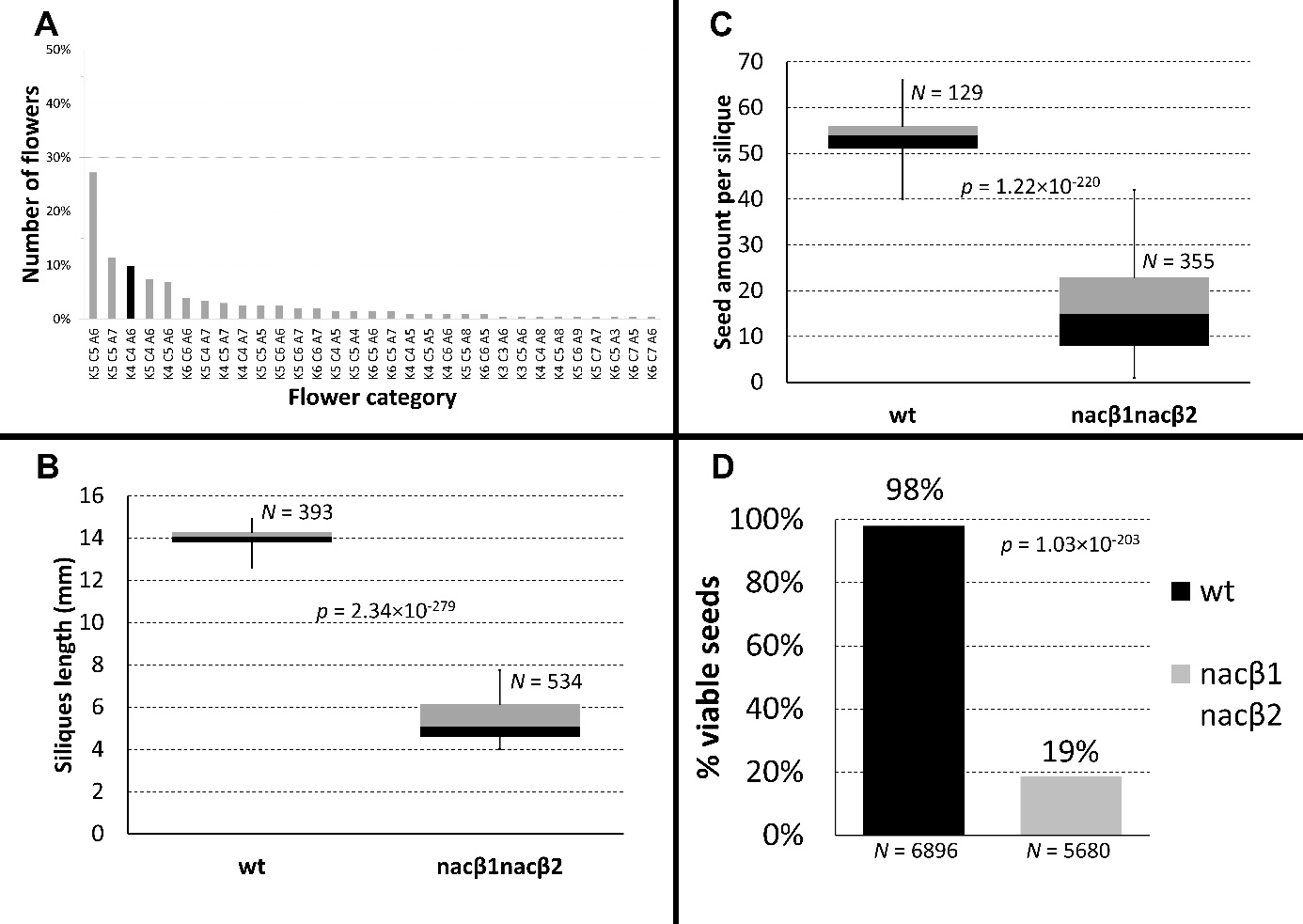
**

**Figure S3 –** **Validation of RNAseq data with qRT-PCR**. Linear regression of log_2_FPKM values obtained from RNA-seq data and cT values from RT-qPCR for each genotype for twelve DE genes in *nacβ1nacβ2 (*At1g17880, At1g73230, At2g20142, At5g64120, At2g43510, At3g12580, At5g52390, At3g12390, At3g49470, At5g13850, At4g10480, At1g33040) with correlation coefficient R^2^ = 0.7842.

**
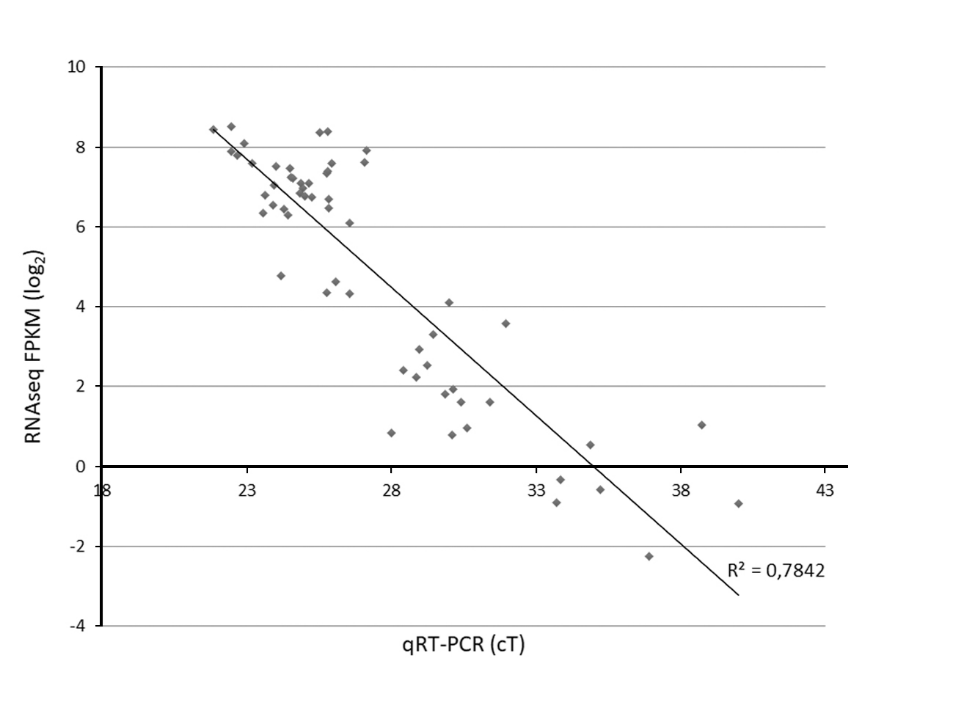
**

**Figure S4 – Germination of *nacβ1nacβ2* under salt and osmotic stress.** A – Germination rate of Col-0 wt compared to *nacβ1nacβ2* under salt stress caused by 50 mM, 100mM, 125 mM, and 150 mM NaCl. Error bars represent ±5%. The calculations of germinated seeds were performed 4, 6, 8, 11, and 13 days after seed sowing. B – Germination rate of Col-0 wt compared to *nacβ1nacβ2* under osmotic stress caused by 250 mM, 300mM, and 350 mM mannitol. Error bars represent ±5%. The calculations of germinated seeds were performed 4, 6, 8, 11, and 13 days after seed sowing. C – A representative plate of seedlings 13 days after sowing on 125 mM NaCl. D – A representative plate of seedlings 13 days after sowing on 150 mM NaCl. E - A representative plate of seedlings 13 days after sowing under control conditions without added stress agent.


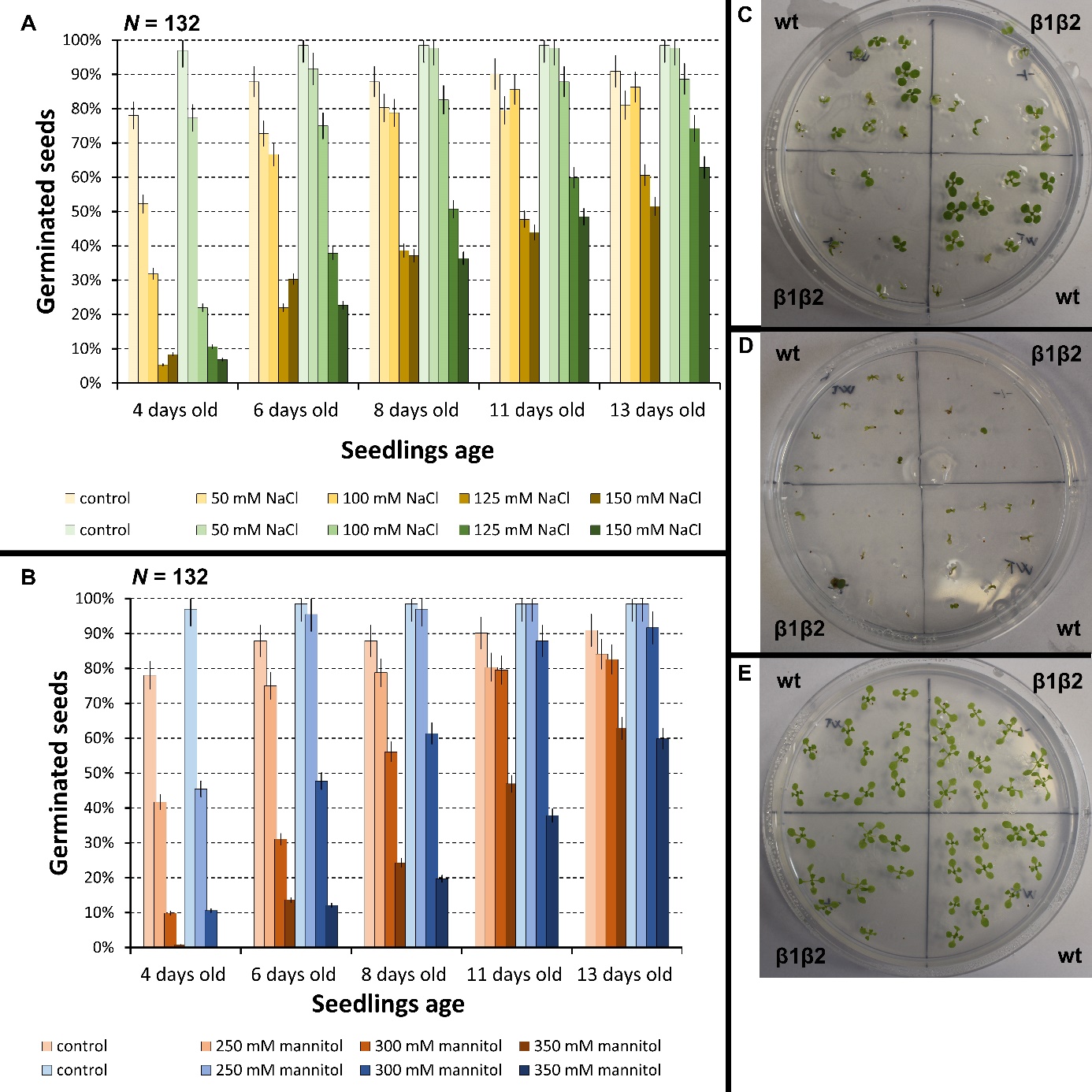

Supplement: Supplementary file 1 [file ijms-21-02065-s001.zip › supplementary_files.docx]
