# Supplementary figures and images for: The Beta Subunit of Nascent Polypeptide Associated Complex Plays A Role in Flowers and Siliques Development of Arabidopsis thaliana
Source: Int J Mol Sci. 2020 Mar 17;21(6):2065. doi: 10.3390/ijms21062065 (PMC7139743; doi:10.3390/ijms21062065)

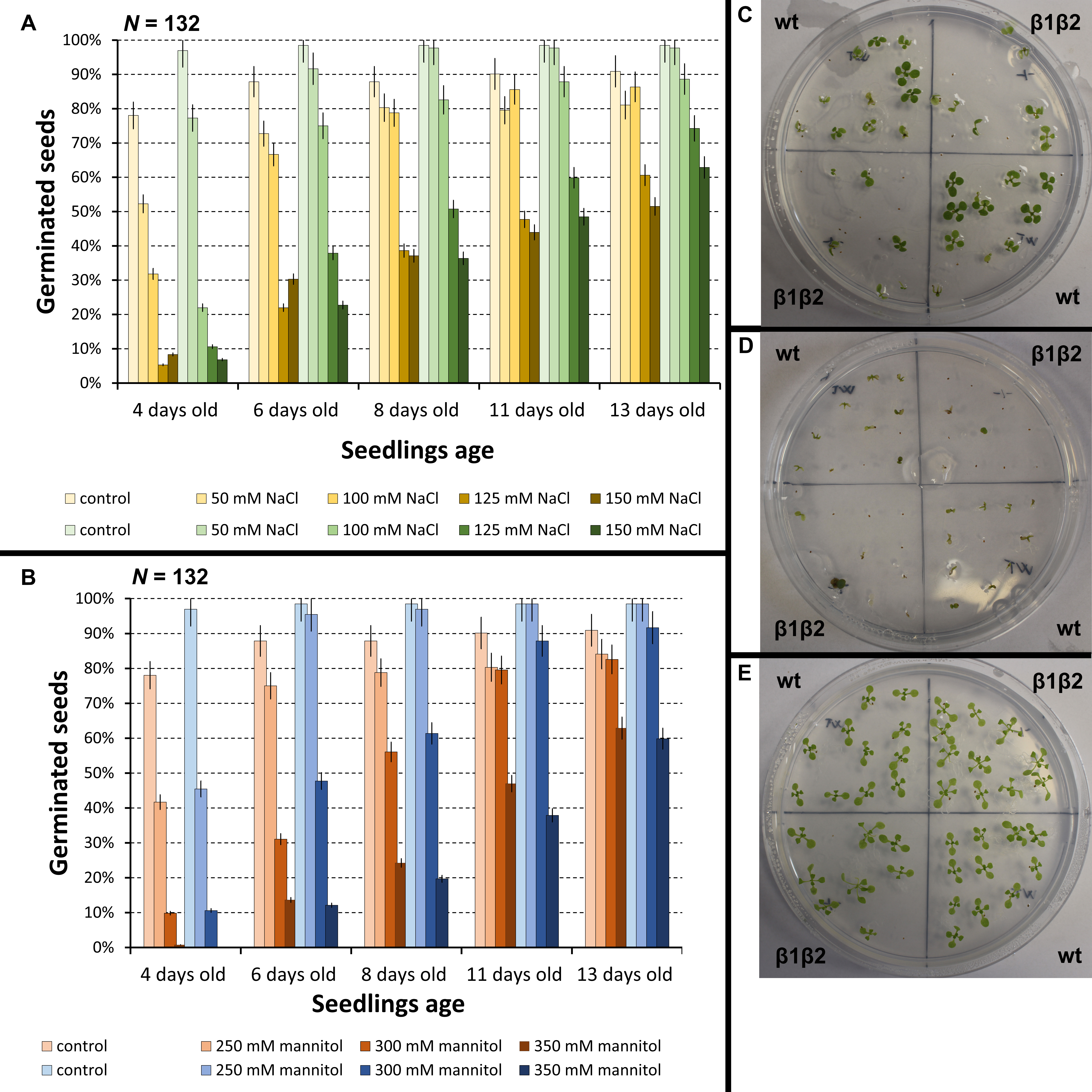

Supplement: Supplementary file 1 [file ijms-21-02065-s001.zip › figS4.jpg]

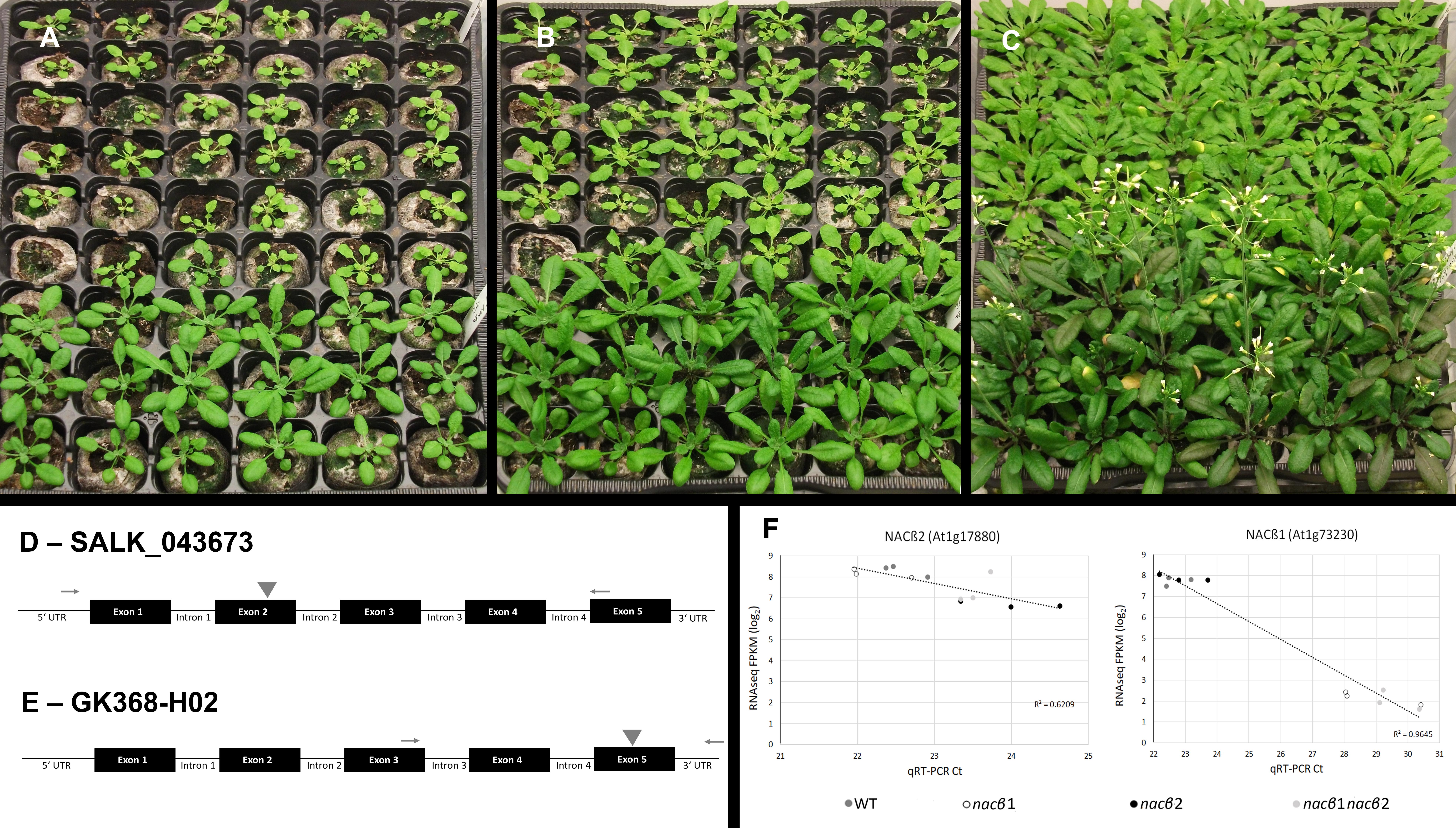

Supplement: Supplementary file 1 [file ijms-21-02065-s001.zip › figS1_new.jpg]

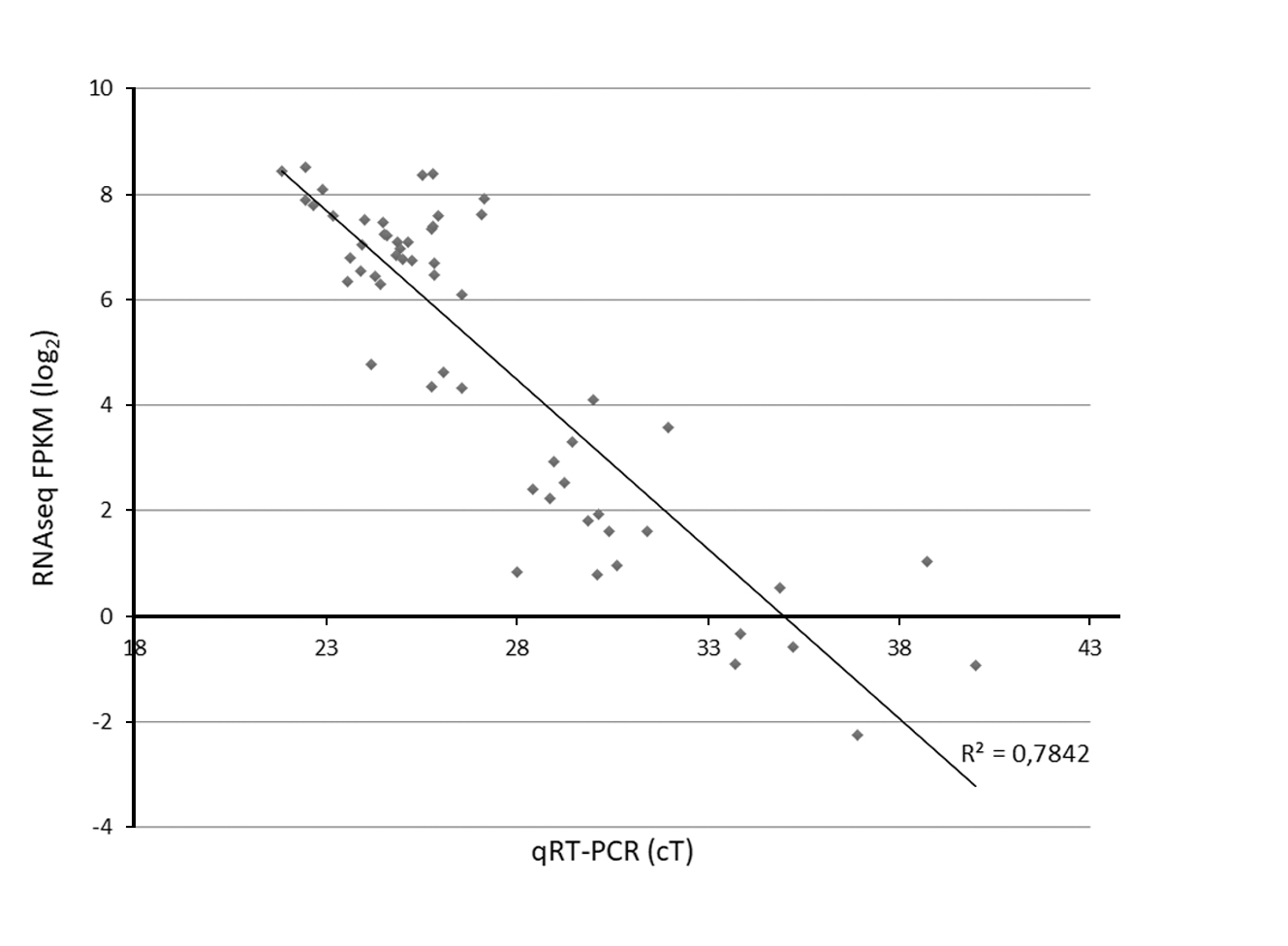

Supplement: Supplementary file 1 [file ijms-21-02065-s001.zip › figS3.tif]
